# Aqueous reductive amination using a dendritic metal catalyst in a dialysis bag

**DOI:** 10.3762/bjoc.9.110

**Published:** 2013-05-17

**Authors:** Jorgen S Willemsen, Jan C M van Hest, Floris P J T Rutjes

**Affiliations:** 1 Radboud University Nijmegen, Institute for Molecules and Materials, Heyendaalseweg 135, 6525 AJ Nijmegen, The Netherlands

**Keywords:** aqueous reductive amination, cascade catalysis, compartmentalization, dendritic catalysts, dialysis

## Abstract

Water-soluble dendritic iridium catalysts were synthesized by attaching a reactive metal complex to DAB-Am dendrimers via an adapted asymmetric bipyridine ligand. These dendritic catalysts were applied in the aqueous reductive amination of valine while contained in a dialysis bag. Comparable conversions were observed as for the noncompartmentalized counterparts, albeit with somewhat longer reaction times. These results clearly show that the encapsulated catalyst system is suitable to successfully drive a complex reaction mixture with various equilibrium reactions to completion.

## Introduction

Cascade catalysis, a bioinspired strategy to conduct multiple consecutive catalytic steps in one pot, is attracting the attention of an increasing number of chemists [1–9]. Advantages of cascade processes include a reduction in the number of workup and purification steps, but also the fact that unstable intermediates can be immediately further reacted, or that unfavorable equilibria can be driven to the desired product. An obvious drawback of such a strategy may be the often intrinsic incompatibility of the catalysts and/or enzymes involved, which will lead to incomplete conversions. Incompatible catalysts can be physically separated in various ways, e.g., by applying biphasic reaction conditions [10], membrane reactors [11] or sol–gels [12]. Another way to circumvent incompatibility problems is to achieve compartmentalization by attaching the actual catalyst to larger particles and contain them in an environment that is accessible for the substrate molecules, but impermeable to the macromolecular catalyst. Such “pseudo-homogeneous catalysts” can, amongst other methods, be created by immobilizing soluble metal complexes on nanoparticles, either nonmagnetic [13–14] or magnetic ones [15–16], which can be contained in semipermeable membranes and hence be physically separated from other catalysts. Nanoparticles, however, may be unstable, and stabilizers, which may affect the catalyst behavior, are often necessary to prevent aggregation [17–18].

**Figure 1:**
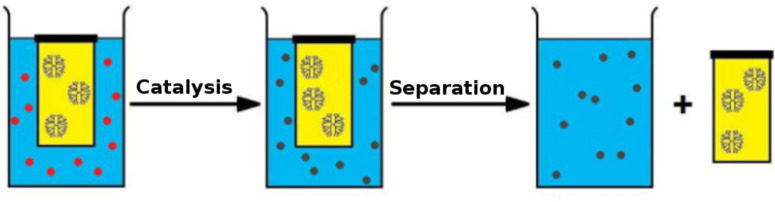
General approach for the use of dendritic catalysts in a dialysis bag.

Dendrimers can also be used as scaffolds for creating macromolecular catalysts [19], which generally show only a limited loss in activity compared to their “nonexpanded” congeners [20–21]. Such dendritic catalysts are kept in a compartment due to the macromolecular nature of these polymers, and have been utilized for purification purposes after the reaction [22] and in continuous-flow reactors during the reaction [23]. Dendritic catalysts have also been applied while enclosed in commercially available dialysis bags [24–26]. The latter examples, however, were conducted in organic, environmentally unfriendly solvents. As part of a research program, in which we focus on conducting cascade reactions catalyzed by the joint action of organometallic catalysts and enzymes, we studied possibilities to compartmentalize metal catalysts in an aqueous environment. Inspired by the aforementioned dendrimer examples, we decided to design a metallodendrimer that would show catalytic activity in a cascade process while compartmentalized under aqueous conditions. To this end, an iridium catalyst was selected that was known to be suitable for reductive amination in an aqueous environment. We showed that by applying a dendritic analogue in a dialysis device we were able to successfully drive the aqueous reductive amination of an unprotected amino acid to completion, despite the unfavorable equilibria for iminium ion formation in water. Since the dendrimer remains inside the dialysis bag due to its size, the catalyst can also be easily removed from the reaction mixture after the reaction has been completed (Figure 1).

## Results and Discussion

**Scheme 1:**
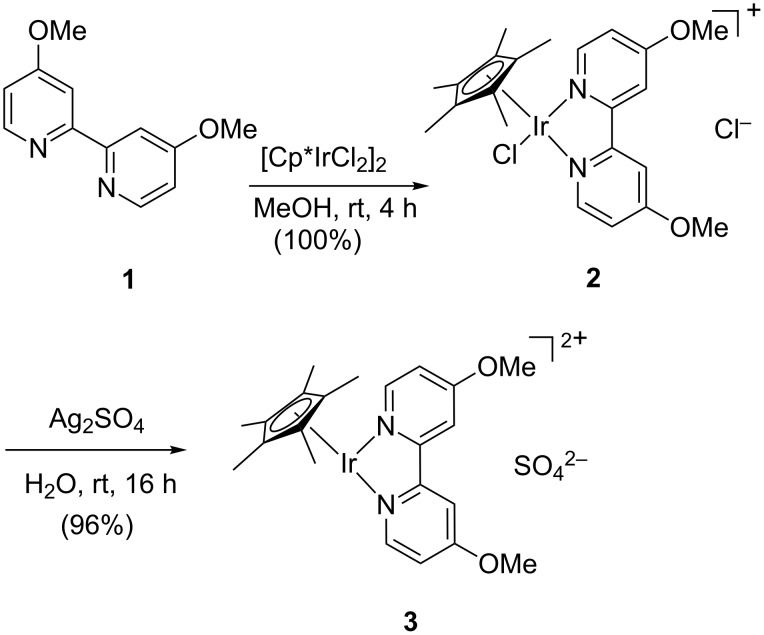
Synthesis of water-soluble iridium catalyst **3**.

To study this approach, we selected iridium catalyst **3**, which has been previously successfully applied in aqueous reductive aminations [27]. The catalyst is based on related iridium complexes that are capable of performing transfer hydrogenation reactions, as has been documented by Fukuzumi et al. [28–30]. The water-soluble iridium catalyst **3** was prepared according to a procedure of Francis in high yield (Scheme 1) [27]. The starting material is [Cp*IrCl_2_]_2_ which readily coordinates to bipyridine **1** to form iridium complex **2**. Subsequent abstraction of the chloride ion in the presence of Ag_2_SO_4_ afforded the active species **3** in excellent yield.

**Scheme 2:**
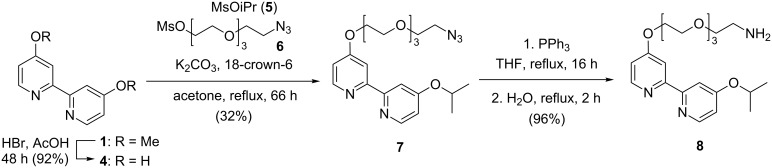
Synthesis of the desymmetrized bipyridine **8**.

To attach catalyst **3** to a dendrimer, an amine functionality was introduced on the bipyridine ligand in three steps (Scheme 2). The first step involved demethylation of 4,4’-dimethoxy-2,2’-bipyridine (**1**) in 92% yield [31]. Monofunctionalization of the ligand will lead to the highest possible catalyst loading per dendrimer and also prevents cross-linking between dendrimers. Therefore, a 1:1 mixture of isopropyl mesylate (**5**) and tetraethylene glycol azido mesylate **6** was reacted with diol **4**, affording a mixture of the desired asymmetric bipyridine **7** and the two corresponding symmetric bipyridines. This mixture was separated by column chromatography resulting in 32% isolated yield of pure azide **7**. Subsequent Staudinger reduction and purification by acid–base extraction afforded the amine-containing ligand **8** in 96% yield.

**Scheme 3:**
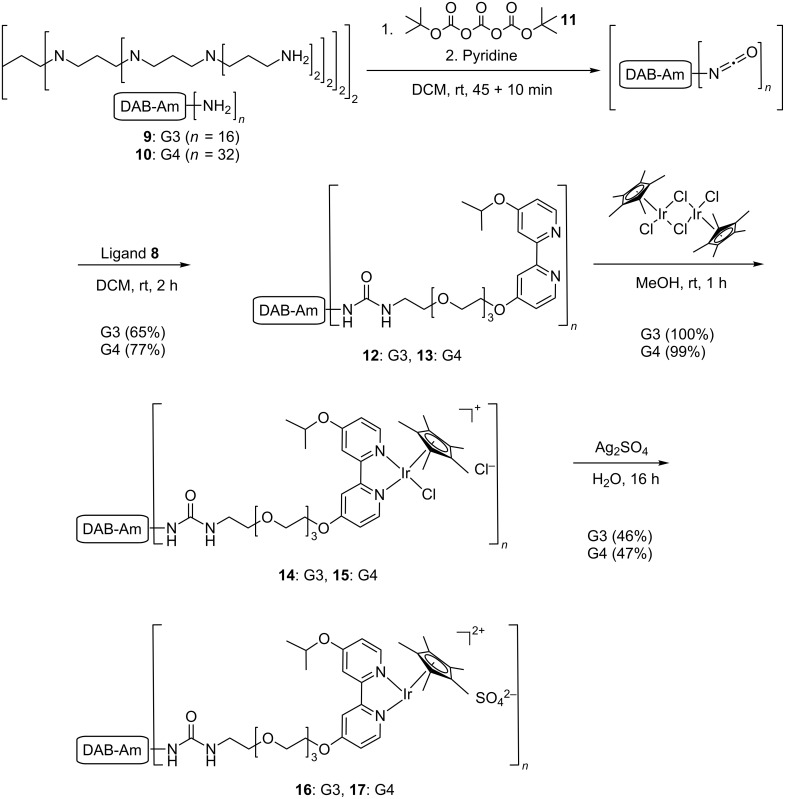
Attachment of the adapted ligand **8** to the dendrimers via a multi-isocyanate coupling, followed by iridium coordination.

Ligand **8** was connected to water-soluble DAB-Am dendrimers via a protocol described by Peerlings and Meijer (Scheme 3) [32]. First, a multi-isocyanate was prepared in situ by using di-*tert*-butyl tricarbonate (**11**)*.* After the addition of pyridine to quench the excess of tricarbonate **11**, ligand **8** was added, leading to the formation of dendrimers **12** and **13** in good yields. The products were completely characterized by ^1^H and ^13^C NMR, thereby confirming that all amines had been reacted. Furthermore, IR spectroscopy showed signals at 1580 and 1640 cm^−1^ derived from the newly formed urea functionalities. The next reaction, coordination of iridium to the dendritic ligands in methanol, afforded the corresponding iridium complexes **14** and **15** in quantitative yield. In the final step, the complex was converted into the water-soluble dendritic catalysts **16** and **17** by overnight treatment with Ag_2_SO_4_ in 46 and 47% yield, respectively. During the removal of AgCl by centrifugation, some dendritic material was probably lost in the pellet, explaining the rather moderate yield of the final step. Dendrimers **16** and **17** were purified by aqueous dialysis. Unfortunately, the NMR spectra of dendrimers **14**–**17** could not be completely resolved due to the broad peaks observed as well as the low intensity of the resonances in the ^13^C NMR measurements. However, each transformation was accompanied by a clear and complete change in chemical shift of the aromatic bipyridine peaks.

**Scheme 4:**
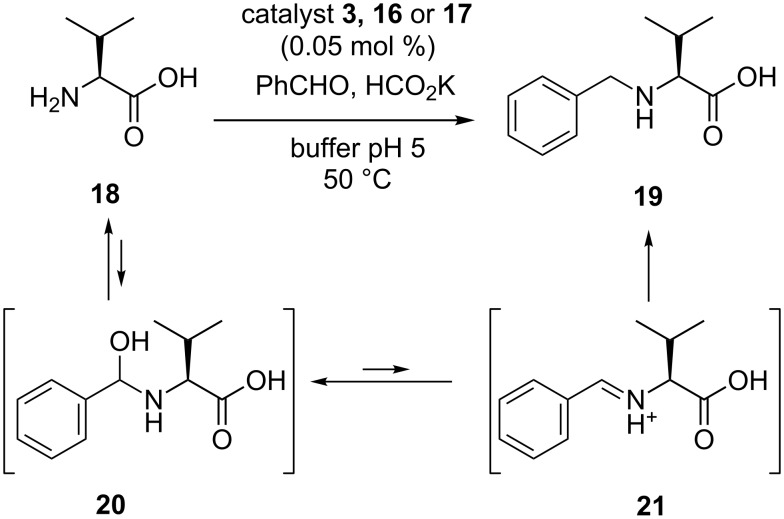
Catalytic reductive amination of valine (**18**) via unfavorable equilibrium reactions in water.

The reactivity of macromolecular catalysts **16** and **17** was examined in the aqueous reductive amination of valine (**18**, Scheme 4). This reaction is in fact a multistep process, in which the equilibria unfavorable in water for the formation of hemiaminal **20** and iminium ion **21** are compensated by the iridium-catalyzed reduction to form the benzylated amino acid **19**. Iridium complexes **3**, **16** and **17** were capable of reducing the intermediate imine in the presence of HCO_2_K, which acted as the hydride source. The reaction was monitored by taking aliquots of the reaction mixture and by HPLC analysis. An excess of benzaldehyde was used, because it was also partially reduced to benzyl alcohol in a side reaction. The optimal pH appeared to be 5, comparable with a similar catalyst published by Fukuzumi [29–30].

**Figure 2:**
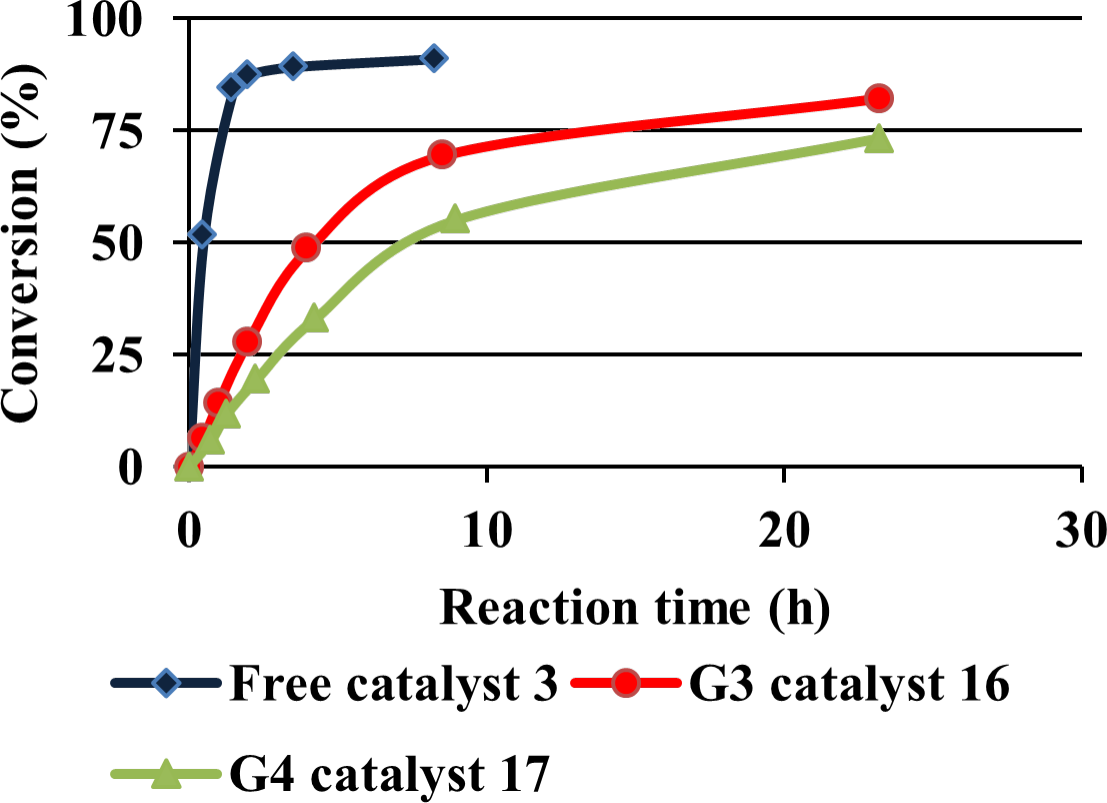
Formation of **19** catalyzed by the three iridium catalysts.

In the presence of the nondendritic catalyst **3**, formation of the N-alkylated amino acid **19** proceeded considerably faster than with the dendrimer-supported reductive aminations (Figure 2). However, after longer reaction times the yields were only slightly lower for the dendritic catalysts.

**Figure 3:**
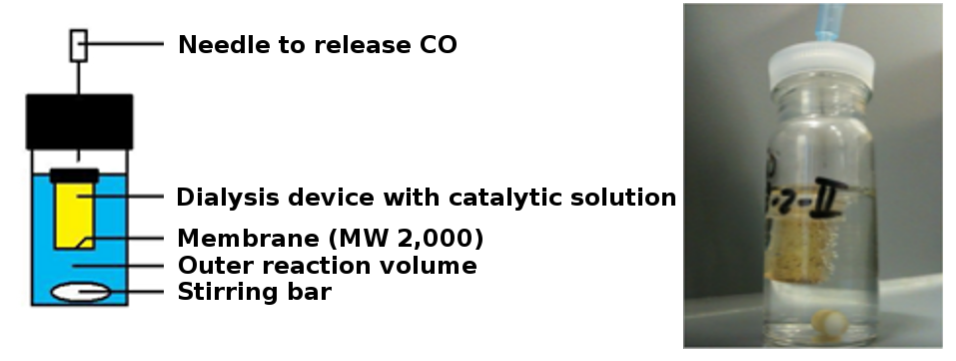
Reaction setup to perform compartmentalized catalysis.

**Figure 4:**
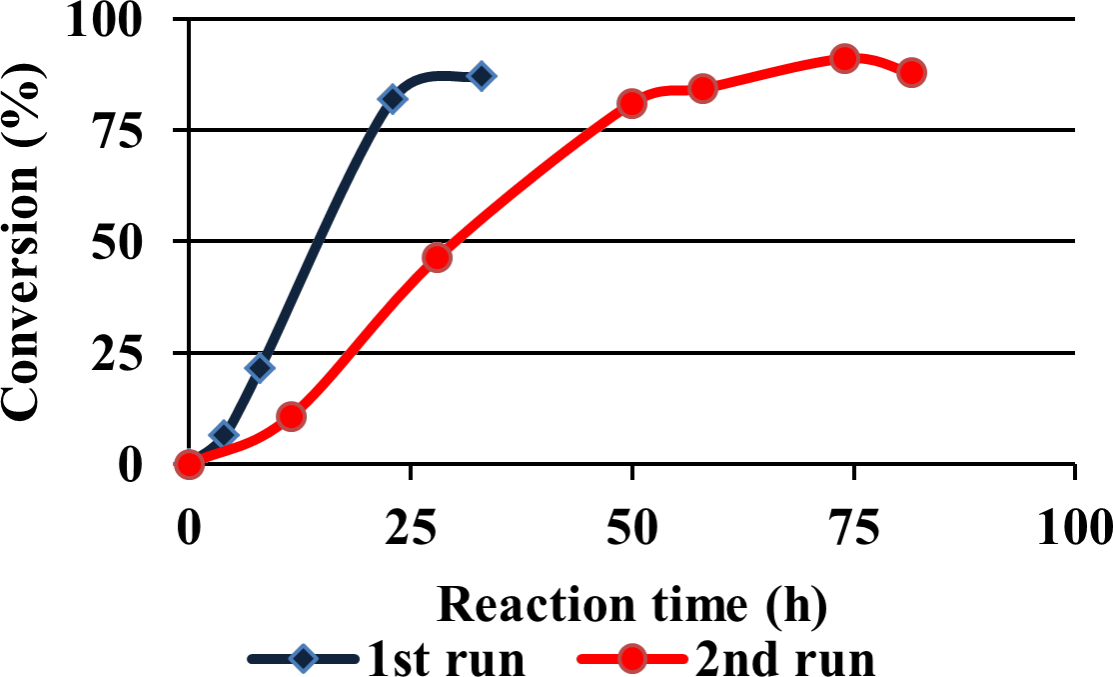
G3 catalyst **16** activity in dialysis device.

In order to study the same reaction with the dendritic catalysts **16** and **17** in a dialysis bag, a different setup was used based on commercially available materials. This consisted of a cup which could be closed and contained a dialysis membrane with a molecular-weight cut-off value of 2,000. We assumed that all reaction components except the catalyst would be able to pass through the membrane, turning the dialysis tube into a compartment suitable for catalysis. To verify this hypothesis, catalyst **16** was injected in the dialysis device and the reductive amination of valine was performed under otherwise identical conditions (Figure 3). HPLC analysis showed that the reaction rate and conversion were fairly similar to the noncompartmentalized aqueous reaction (Figure 4), demonstrating that mass transport of the small substrate and product molecules through the membrane is hardly rate-limiting.

After the appropriate reaction time, the dialysis tube was removed from the reaction mixture, dialyzed in buffer to remove all the other reaction contents and added to a fresh reaction mixture. In the second run, the recovered G3 dendrimer **16** still showed catalytic activity (Figure 4), albeit that a distinct decrease in reaction rate was observed. This phenomenon may be attributed to leakage of the catalyst through the dialysis membrane.

**Figure 5:**
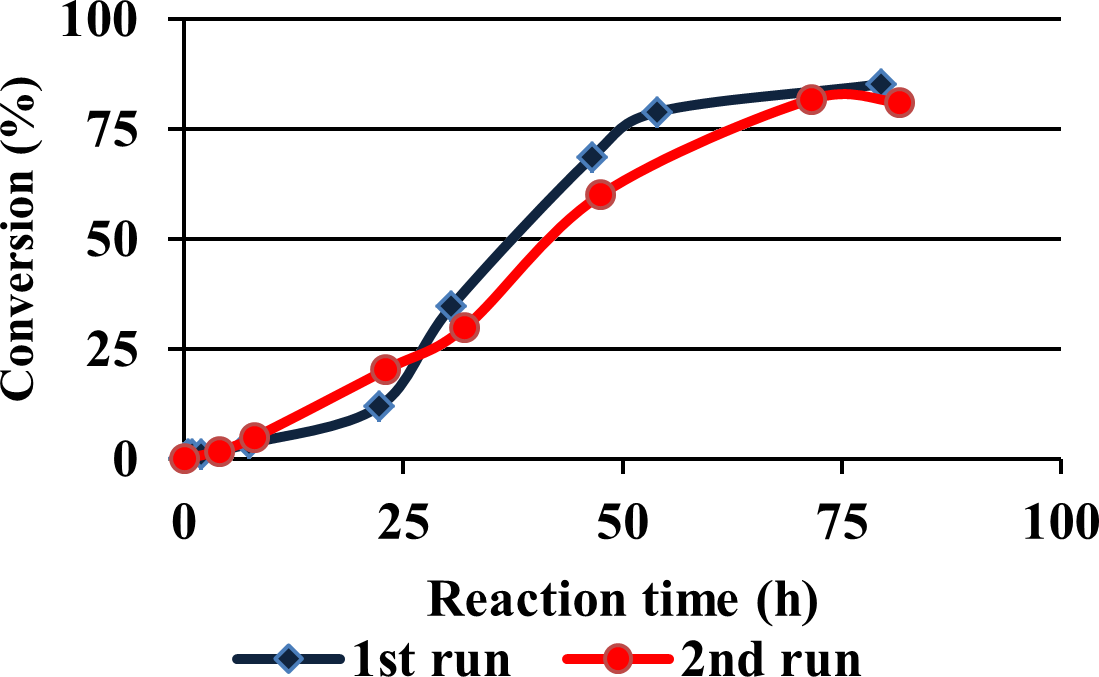
G4 catalyst **17** activity in subsequent runs.

Similar experiments were conducted with the G4 dendrimer **17** (Figure 5). The figure shows that the reaction rate is somewhat lower, but that the conversion is fairly similar to that for the free dendritic catalyst. In contrast to the smaller G3 dendrimer **16**, however, the G4 catalyst **17** was equally active in a second run, clearly demonstrating the viability of containment of the catalyst in the dialysis bag. This result also suggests that the immobilized catalyst should be applicable in cascade reactions with reagents that are normally incompatible with the iridium complex. Since our process was successfully conducted in water, combinations with enzymes should also be possible.

## Conclusion

Dendritic catalysts were successfully synthesized by connecting Ir-bipyridine complexes to third and fourth generation DAB-Am dendrimers. The dendritic catalysts showed good activity in the multistep reductive amination of a free amino acid in water, albeit that prolonged reaction times as compared to the free catalyst were required. Due to the size of the macromolecular catalysts, it was possible to employ them in a dialysis device for conducting reductive aminations of free amino acids in water. The G4 catalyst showed the same reaction rate in a second run, which validates the concept of maintaining the macromolecular catalyst in the compartment. The work described here could be more widely applicable for compartmentalization of catalytic systems in aqueous media, where the iridium system may be used either in combination with other catalysts, or with enzymes. These topics are currently under investigation in our group.

## Supporting Information

File 1 Experimental details and spectroscopic data.

File 2 Spectra of compounds.
