# Supplementary material for: Aqueous reductive amination using a dendritic metal catalyst in a dialysis bag
Source: Beilstein J Org Chem. 2013 May 17;9:960–5. doi: 10.3762/bjoc.9.110 (PMC3678615; doi:10.3762/bjoc.9.110)
Supplement: File 1 — Experimental details and spectroscopic data. [file Beilstein_J_Org_Chem-09-960-s001.pdf]

Supporting Information File 1

for

Aqueous reductive amination using a dendritic metal catalyst in a dialysis bag

Jorgen S. Willemsen, Jan C. M. van Hest* and Floris P. J. T. Rutjes*

Address: Radboud University Nijmegen, Institute for Molecules and Materials, Heyendaalseweg 135, 6525 AJ Nijmegen, The Netherlands

Email: Jan C. M. van Hest - J.vanHest@science.ru.nl, Floris P. J. T. Rutjes - F.Rutjes@science.ru.nl

* Corresponding author

Experimental details and spectroscopic data

General information: Solvents were distilled from appropriate drying agents prior to use and stored under nitrogen. Chemicals were purchased from Sigma-Aldrich and used as received. DAB-Am-16 (**9**) and DAB-Am-32 (**10**) were kindly donated by SyMO-Chem. All reactions except the aqueous reductive aminations were carried out under an inert atmosphere of dry argon. Reactions were followed with thin layer chromatography (TLC) on silica-gel-coated plates (Merck 60 F254). IR spectra were recorded on a Thermo Mattson IR 300 FTIR spectrometer. NMR spectra were recorded on a Bruker DMX 300 (300 MHz), a Varian 400 (400 MHz) or a Bruker Avance III 500 (500 MHz) spectrometer. Chemical shifts are given in ppm with respect to tetramethylsilane (TMS) as internal standard. Coupling constants are reported as *J*-values in Hz. Flash column chromatography was carried out using Silicycle SilicaFlash P60 gel (40–63 µm). High-resolution mass spectra were recorded on a JEOL AccuTOF (ESI) or a MAT900 (EI, CI, and ESI). Dendrimers **12** and **13** were purified with Spectrumlabs Spectra/Por Dialysis Membrane 2 with a MWCO of 3.500. The aqueous reductive aminations were performed in Slide-A-Lyzer MINI dialysis units of Thermo Scientific with a MWCO of 2.000. The reductive aminations were monitored with an Agilent 1120 Compact LC equipped with an Agilent Eclipse Plus C18 column. Yields were determined using a calibration curve.

General procedure for the reductive amination of valine: The appropriate catalyst (0.05 equiv), L-valine (0.50 mg, 4.27 µmol) and HCO_2K (3.59 mg, 43 µmol, 10 equiv) were added via a stock solution to citric acid/Na citrate 25 mM buffer pH 5.0. Benzaldehyde (4.34 µL, 43 µmol, 10 equiv) was directly added. The total reaction volume was 1.000 mL. The mixture was stirred at 50 °C open to the atmosphere.

General procedure for the reductive amination in a dialysis cup: A solution of the catalyst (0.05 equiv) in 0.500 mL citric acid/Na citrate 25 mM buffer pH 5.0 was added to the dialysis cup, while L-valine (5.00 mg, 42.7 μmol), HCO_2K (35.9 mg, 0.427 mmol, 10 equiv) and benzaldehyde (43.4 μL, 0.427 mmol, 10 equiv) were added to 9.500 mL citric acid/Na citrate 25 mM buffer pH 5.0. The dialysis cup was placed in this solution. The mixture was stirred at 50 °C open to the atmosphere.

Cp*Ir(4,4'-dimethoxy-2,2'-bipyridine)Cl_2 (2): The procedure described by Francis [1] was used and yielded 100% of the desired product. To a solution of pentamethylcyclopentadienyliridium(III) chloride dimer (100 mg, 0.126 mmol, 0.5 equiv) in MeOH (12 mL) was added 4,4'-dimethoxy-2,2'-bipyridine (**1**) (54 mg, 0.252 mmol). The mixture was stirred for 4 h, followed by evaporation of the volatiles under reduced pressure. The residue was dissolved in as the smallest amount of DCM possible. The product precipitated after the addition of heptane. Filtration and washing with heptane afforded 155 mg (0.252 mmol, 100%) of **2** as a yellow solid. ^1H NMR (400 MHz, $CDCl_3$): δ 9.25 (d, *J* = 2.7 Hz, 2H), 8.37 (d, *J* = 6.5 Hz, 2H), 7.08 (dd, *J* = 6.5, 2.7 Hz, 2H), 4.42 (s, 6H), 1.65 (s, 17H). ^{13}C NMR (75 MHz, $CDCl_3$): δ 169.36, 158.17, 149.94, 117.34, 111.52, 88.14, 77.16, 59.55, 8.86, 1.16.

Cp*Ir(4,4'-dimethoxy-2,2'-bipyridine)SO_4 (3): The procedure described by Francis [1] was used and afforded 96% of the desired compound. To complex **2** (165 mg, 0.27 mmol) in 20 mL water was added Ag_2SO_4 (85 mg, 0.27 mmol, 1.01 equiv). The mixture was stirred at ambient temperature overnight. The mixture was filtered and concentrated under reduced pressure, yielding 165 mg (0.26 mmol, 96%) of a yellow solid. ^1H NMR (400 MHz, D_2O): δ 8.88 (d, *J* = 6.6 Hz, 2H), 7.97 (d, *J* = 2.7 Hz, 2H),

7.41 (dd, J = 6.6, 2.7 Hz, 2H), 4.11 (s, 6H), 1.66 (s, 15H). ^{13}C NMR ($CDCl_3$, 75 MHz): δ = 168.44, 156.88, 151.81, 114.10, 110.15, 87.68, 56.35, 7.21.

4,4'-Dihydroxy-2,2'-bipyridine (4): An adapted procedure was used as described by Gorman [2]. To a solution of 4,4'-dimethoxy-2,2'-bipyridine (**1**) (5.0 g, 23.1 mmol) in 400 mL of a 3:1 AcOH/H_2O mixture was added HBr (33% in AcOH, 40.5 mL, 231 mmol, 10 equiv). The mixture was heated under reflux for 47 h. The mixture was allowed to cool to room temperature and concentrated in vacuo. The residue was dissolved in 250 mL water and the acidic solution was basified by adding a 25% ammonium hydroxide solution. A white precipitate was formed, which was removed by filtration. Adding more hydroxide to the suspension led to a clear solution. Addition of aqueous concentrated HCl led to a white precipitate, which was filtered and combined with the first filtrate. The product was dried under high vacuum, affording 3.99 g (23.1 mmol, 92%) of a white powder. ^{1}H NMR (D_2O + NaOH, 300 MHz): δ = 7.96–7.85 (m, 2H), 6.88–6.93 (m, 2H), 6.38 (dd, J = 5.7, 2.1 Hz, 2H). ^{13}C NMR (D_2O + NaOH, 75 MHz): δ = 174.4, 156.9, 149.0, 115.0, 112.7. IR (ATR): 3209 (br), 3044 (br), 2859, 2772, 1597, 1523, 1467, 1402, 1225 cm^{-1}.

Isopropyl mesylate (5): This material was prepared in 94% yield according to a literature procedure [3]. To a mixture of iPrOH (10.0 mL, 131 mmol, 1.5 equiv) and Et_3N (12.1 mL, 87 mmol, 1 equiv) in 75 mL DCM was added MsCl (6.76 mL, 87 mmol) at 0 °C. The mixture was allowed to warm to ambient temperature and stirred for 2 h. DCM was added to the mixture and the product washed with a 1 M HCl solution. The aqueous phase was extracted once with DCM. The combined organic extracts were washed with brine, dried over $MgSO_4$ and concentrated under

reduced pressure, affording 11.4 g of a yellow oil (82 mmol, 94%). ^{1}H NMR (300 MHz, $CDCl_3$): δ = 4.92 (hept, *J* = 6.2 Hz, 1H), 2.96 (s, 3H), 1.39 (d, *J* = 6.3 Hz, 6H). ^{13}C NMR (75 MHz, $CDCl_3$): δ = 76.82, 38.78, 23.05. IR (ATR): 2984, 2941, 1342, 1324, 1173, 1095, 914, 879, 810 cm^{-1}.

Tetra(ethyleneglycol) azido mesylate 6: A solution of tetraethylene glycol (17.8 mL, 103 mmol) in 200 mL THF was cooled to 0 °C, followed by the dropwise addition of MsCl (8.37 mL, 108 mmol, 1.05 equiv) in 20 mL THF and Et_3N (40 mL, 288 mmol, 2.8 equiv). The mixture was allowed to warm to room temperature and stirred for 2 h. The reaction mixture was filtered and concentrated in vacuo, yielding a colorless oil. The product was dissolved in 400 mL MeOH, and NaN_3 (33.5 g, 515 mmol, 5 equiv) was added. The reaction mixture was heated under reflux overnight. The mixture was allowed to cool to room temperature, filtered and concentrated in vacuo. Brine was added, and the product was extracted with DCM (4 ×). The organic fractions were dried over $MgSO_4$ and concentrated under reduced pressure. The product was purified with column chromatography on silica (DCM/MeOH 1:99 to 4:96), yielding 9.16 g (41.8 mmol, 41%) of a colorless oil as the pure desired intermediate (**6a**). ^{1}H NMR (300 MHz, $CDCl_3$): δ = 3.69 (m, 2H), 3.66–3.62 (m, 10H), 3.60–3.54 (m, 2H), 3.39–3.32 (m, 2H), 2.79 (br s, 1H). ^{13}C NMR (75 MHz, $CDCl_3$): δ = 72.49, 70.64, 70.61, 70.54, 70.30, 69.99, 61.62, 50.62. IR (ATR): 3420 (br), 2863, 2098, 1290, 1234, 1095, 931 cm^{-1}.

To a solution of tetra(ethyleneglycol) azide (**6a**) (9.16 g, 41.8 mmol) in 160 mL THF was added dropwise a solution of MsCl (4.20 mL, 54.3 mmol, 1.3 equiv) in 20 mL THF at 0 °C. Next, a solution of Et_3N (21.5 mL, 155 mmol, 3.7 equiv) in 20 mL THF

was added dropwise. The mixture was allowed to warm to room temperature and stirred for 2.5 h. Solids were removed by filtration and the mixture concentrated under reduced pressure. The residue was dissolved in DCM and washed with a 1 M HCl solution, and the acidic layer was extracted with DCM. The combined organic fractions were washed with brine, dried over $MgSO_4$ and concentrated under reduced pressure, yielding 12.4 g (41.7 mmol, 100%) of a yellow oil. ^{1}H NMR (300 MHz, $CDCl_3$): δ = 4.41–4.33 (m, 2H), 3.80–3.73 (m, 2H), 3.71–3.61 (m, 10H), 3.44–3.32 (m, 2H), 3.06 (s, 3H). ^{13}C NMR (75 MHz, $CDCl_3$): δ = 77.16, 70.70, 70.66, 70.08, 69.37, 69.05, 50.73, 37.72. IR (ATR): 2919, 2872, 2098, 1355, 1173, 1104, 931, 797 cm^{-1}.

4-(Tetra(ethyleneglycol) azido 4'-isopropoxy-2,2'-bipyridine (7): To a solution of iPrOMs (**5**) (1.42 g, 10.3 mmol, 1.05 equiv), tetra(ethyleneglycol) azido mesylate (**6**) (3.05 g, 10.3 mmol, 1.05 equiv) in 110 mL acetone was added 2,2'-bipyridine-4,4'-diol (1.84 g, 9.77 mmol), K_2CO_3 (3.38 g, 24.4 mmol, 2.5 equiv) and 18-crown-6 (258 mg, 0.98 mmol, 0.10 equiv). The mixture was heated under reflux for 66 h. The mixture was filtered and the solvent removed. Saturated $NaHCO_3$ solution was added and the product extracted with DCM (2 ×). The organic extracts were washed with brine, dried over $MgSO_4$ and concentrated under reduced pressure. The compound was purified by column chromatography on silica (DME/DCM, 2:98 to 10:90). The fractions were analyzed by LRMS and only the pure ones were selected, yielding 1.34 g (3.10 mmol, 32%) of the desired product as a yellow oil. ^{1}H NMR (400 MHz, $CDCl_3$): δ = 8.44 (dd, J = 7.8, 5.7 Hz, 2H), 7.94 (dd, J = 10.6, 2.5 Hz, 2H), 6.85 (dd, J = 5.7, 2.6 Hz, 1H), 6.79 (dd, J = 5.7, 2.6 Hz, 1H), 4.80 (hept, J = 6.0 Hz, 1H), 4.34–4.27 (t, J = 4.8 Hz, 2H), 3.94–3.84 (t, J = 4.4 Hz, 2H), 3.79–3.59 (m, 10H), 3.37 (t, J =

5.1 Hz, 2H), 1.39 (d, *J* = 6.1 Hz, 6H). ^{13}C NMR (75 MHz, $CDCl_3$): δ = 165.95, 165.23, 158.14, 157.88, 150.34, 150.27, 112.17, 111.51, 107.65, 106.74, 77.16, 71.07, 70.85, 70.17, 70.13, 69.52, 67.59, 50.82, 21.98. IR (ATR): 2971, 2872, 2098, 1584, 1549, 1454, 1290, 1229, 1104, 996, 940 cm^{-1}. HRMS (ESI): calcd. for $C_{21}H_{30}N_5O_5$ $[M]^+$ 432.2247; found 432.2257.

4-(Tetra(ethyleneglycol) amino 4'-isopropoxy-2,2'-bipyridine (8): To 4-(tetra(ethyleneglycol) azido 4'-isopropoxy-2,2'-bipyridine **7** (1.14 g, 3.10 mmol) in 40 mL THF was added PPh_3 (1.02 g, 3.87 mmol, 1.25 equiv). The mixture was heated under reflux for 2.5 h, water (2.8 mL) was added, and the mixture heated under reflux for 1 h. The mixture was allowed to cool to room temperature and concentrated in vacuo. A 0.25 M HCl solution was added to the mixture and the aqueous layer was washed with DCM (5 ×). The aqueous phase was basified by adding a 2 M NaOH solution which resulted in a cloudy mixture. The product was extracted six times with DCM. The combined organic extracts were dried over $MgSO_4$ and concentrated in vacuo, giving 1.26 g (2.96 mmol, 96%) of a red oil. ^{1}H NMR (300 MHz, $CDCl_3$): δ = 8.44 (t, *J* = 5.6 Hz, 2H), 7.94 (dd, *J* = 5.4, 2.5 Hz, 2H), 6.86 (dd, *J* = 5.7, 2.6 Hz, 1H), 6.79 (dd, *J* = 5.7, 2.6 Hz, 1H), 4.81 (hept, *J* = 6.0 Hz, 1H), 4.31 (t, *J* = 4.8 Hz, 2H), 3.90 (t, *J* = 4.4 Hz, 2H), 3.79–3.60 (m, 8H), 3.52 (t, *J* = 4.2 Hz, 2H), 2.87 (t, *J* = 5.2 Hz, 2H), 1.96 (br s), 1.39 (d, *J* = 6.1 Hz, 6H). ^{13}C NMR (75 MHz, $CDCl_3$): δ = 165.93, 165.26, 158.12, 157.86, 150.32, 112.19, 111.44, 107.71, 106.82, 77.16, 73.16, 71.06, 70.78, 70.41, 70.16, 69.53, 67.61, 41.82, 21.99. IR (ATR): 3380 (br), 2971, 2867, 1584, 1558, 1458, 1290, 1238, 1113 cm^{-1}. HRMS (ESI): calcd. for $C_{21}H_{32}N_3O_5$ $[M]^+$ 406.2342; found 406.2342.

Di-*tert*-butyl tricarbonate (11): An adapted version of the procedure described by Pope was used [4]. To a solution of potassium *tert*-butoxide (25 mL of a 1.0 M solution in THF, 25 mmol) was added 10 mL THF and through this solution was slowly passed CO_2 at 0 °C for 30 minutes. Diphosgene (0.905 mL, 12.5 mmol, 0.5 equiv) in 8 mL THF was added dropwise at 0 °C and the mixture stirred at 0 °C for 1 h. The mixture was partially concentrated by a rotary evaporator equipped with a cold trap with the reaction flask on an ice bath. Salts were removed by filtration and the residue was extensively rinsed with pentane. A large amount of pentane was removed by rotary evaporation at 0 °C and the mixture cooled to −15 °C. A white product crystallized and was obtained by filtration. The filtrate was removed in vacuo at 0 °C and the white solid that remained was crystallized in pentane, yielding an off-white solid. The pentane was concentrated in vacuo at 0 °C and the remaining solid was again dissolved in a minimal amount of pentane, affording some more off-white solid. The residues were combined, resulting in 1.95 g (7.44 mmol, 60%) of the desired product. ^{1}H NMR (300 MHz, $CDCl_3$): δ = 1.56 (s, 18H). ^{13}C NMR (75 MHz, $CDCl_3$): δ = 144.79, 143.47, 87.42, 27.46. IR (ATR): 1860, 1782, 1069 cm^{-1}.

DAB-G3-(bpy)$_{16}$ 12: To a solution of di-*tert*-butyl tricarbonate (**11**) (90 mg, 0.343 mmol, 1.5 equiv) in 2.5 mL DCM was added dropwise a solution of DAB-Am-16 (**9**) (24.1 mg, 14.29 μmol) in 1.5 mL DCM at room temperature. Gas production was observed. After 45 minutes, a drop of pyridine was added to quench the tricarbonate, followed by the addition of 4-(tetra(ethyleneglycol) amino 4'-isopropoxy-2,2'-bipyridine (**8**) (139 mg, 0.343 mmol, 1.5 equiv) in 2.5 mL DCM after 10 minutes. The mixture was stirred for 2 h and concentrated in vacuo. The mixture was dissolved in a minimal amount of DCM, and heptane was added. The precipitate was removed by

centrifugation. The filtrate was concentrated in vacuo and the procedure repeated, which resulted in some more precipitation. The solid material was dissolved in DCM and concentrated under reduced pressure, giving 80 mg of a sticky white oil (9.31 μmol, 65%). ^{1}H NMR (400 MHz, $CDCl_3$): δ = 8.42 (dd, *J* = 9.2, 5.7 Hz, 32H), 7.92 (dd, *J* = 6.8, 2.3 Hz, 32H), 6.83 (dd, *J* = 5.6, 2.5 Hz, 16H), 6.77 (dd, *J* = 5.6, 2.5 Hz, 16H), 6.20 (br s), 5.94 (br s), 4.83–4.73 (m, 16H), 4.28–4.22 (m, 32H), 3.88–3.82 (m, 32H), 3.76–3.43 (m, 160H), 3.36–3.28 (m, 32H), 3.14 (br s, 32H), 2.46–2.22 (m, 84H), 1.63–1.46 (m, 60), 1.37 (d, *J* = 6.1 Hz, 96H). ^{13}C NMR (75 MHz, $CDCl_3$): δ = 165.88, 165.23, 159.46, 158.04, 157.78, 150.29, 112.15, 111.43, 107.66, 106.75, 70.96, 70.61, 70.28, 70.16, 69.44, 67.54, 52.25, 51.03, 40.07, 38.18, 27.83, 22.27, 22.00, 21.69. IR (ATR): 3347, 2923, 2863, 2789, 1636, 1579, 1562, 1450, 1285, 1108 cm^{-1}.

DAB-G4-(bpy)$_{32}$ (13): The method for the G3 analogue was followed with the same amounts of starting materials, except DAB-Am-32 (**10**) (25 mg, 7.11 μmol), and yielded 95 mg of a sticky white oil (5.48 μmol, 77%). ^{1}H NMR (400 MHz, $CDCl_3$): δ = 8.43–8.40 (m, 64H), 7.94–7.92 (m, 64H), 6.84–6.76 (m, 64H), 6.29 (br s), 6.00 (br s), 4.81–4.75 (m, 32H), 4.31–4.20 (m, 64H), 3.88–3.80 (m, 64H), 3.72–3.53 (m, 256H), 3.52–3.46 (br s, 64H), 2.47–2.21 (br s, 184H), 1.66–1.46 (m, 124H), 1.37 (d, *J* = 6.0 Hz, 192H). ^{13}C NMR (75 MHz, $CDCl_3$): δ = 165.90, 165.24, 159.53, 158.05, 157.80, 150.31, 112.16, 111.44, 107.67, 106.78, 70.97, 70.63, 70.32, 70.17, 69.46, 67.57, 40.07, 38.26, 22.28, 22.02. IR (ATR): 3343, 2928, 2859, 2802, 1640, 1588, 1558, 1281, 1229, 1108 cm^{-1}.

DAB-G3-(Cp*Ir(bpy)Cl$_2$)$_{16}$ (14): To a solution of DAB-G3-(bpy)$_{16}$ (**12**) (60 mg, 6.98 μmol) in 5 mL MeOH was added dichloro(pentamethylpentadienyl)iridium(III) dimer (44.5 mg, 55.8 μmol, 0.5 equiv). The orange mixture turned yellow within a few minutes. After 1 hour, the reaction mixture was concentrated in vacuo. The residue was dissolved in a minimal amount of DCM, and heptane was added until a suspension was formed. The solid was removed by centrifugation, after which the residue was rinsed with heptane, affording 104 mg (6.95 μmol, 100%) of a yellow solid. ^{1}H NMR (400 MHz, CD$_3$OD): δ = 8.69–8.64 (m, 32H), 8.10–7.97 (m, 32H), 7.35–7.25 (m, 32H), 5.02 (hept, *J* = 6.0 Hz, 16H), 4.51–4.31 (m, 32H), 3.95–3.88 (m, 32H), 3.75–3.69 (m, 32H), 3.70–3.65 (m, 32H), 3.65–3.62 (m, 32H), 3.60–3.56 (m, 32H), 3.49–3.43 (m, 32H), 3.26–3.21 (m, 32H), 3.16–3.09 (m, 32H), 2.76–2.50 (m, 84H), 1.70–1.66 (m, 300H) 1.46–1.39 (m, 96H). ^{13}C NMR (75 MHz, CD$_3$OD): δ = 169.03, 168.32, 161.07, 158.33, 158.21, 153.73, 153.61, 117.09, 116.67, 112.63, 111.56, 90.01, 74.06, 71.95, 71.87, 71.66, 71.43, 71.18, 70.56, 70.30, 70.24 52.78, 41.03, 39.17, 21.98, 8.91, 8.68. IR (ATR): 3377 (br), 2936, 2867, 1605, 1562, 1493, 1428, 1229, 1104 cm^{-1}.

DAB-G4-(Cp*Ir(bpy)Cl$_2$)$_{32}$ (15): The method for the G3 analogue was followed with DAB-G4-(bpy)$_{32}$ (**13**) (60 mg, 3.46 μmol) and pentamethylpentadienyliridium(III) chloride dimer (44.2 mg, 55.4 μmol, 0.5 equiv) as starting materials and yielded 103 mg (3.42 μmol, 99%) of a yellow solid. ^{1}H NMR (400 MHz, CD$_3$OD): δ = 8.70–8.64 (m, 64H), 8.10–7.97 (m, 64H), 7.34–7.24 (m, 64H), 5.03 (hept, *J* = 6.0 Hz, 32H), 4.50–4.32 (m, 64H), 3.95–3.88 (m, 64H), 3.76–3.71 (m, 64H), 3.71–3.66 (m, 64H), 3.66–3.61 (m, 64H), 3.61–3.56 (m, 64H), 3.50–3.44 (m, 64H), 3.27–3.21 (m, 64H), 3.17–3.10 (m, 64H), 2.70–2.44 (m, 184H), 1.70–1.66 (m, 604H), 1.47–1.39 (m,

196H). ^{13}C NMR (75 MHz, $CDCl_3$): δ = 168.93, 168.26, 160.98, 158.18, 153.68, 116.90, 116.71, 112.54, 112.44, 89.98, 74.01, 71.80, 71.75, 71.63, 71.52, 71.45, 71.38, 22.39, 22.17, 8.85. IR (ATR): 3381 (br), 2936, 2889, 1614, 1549, 1497, 1445, 1225, 1104 cm^{-1}.

DAB-G3-(Cp*Ir(bpy)SO_4)$_{16}$ (16): To a solution of DAB-G3-(Cp*Ir(bpy)Cl_2)$_{16}$ (**14**) (94 mg, 6.28 µmol) in 5 mL water was added Ag_2SO_4 (31.3 mg, 101 µmol, 1 equiv). The mixture was stirred at ambient temperature overnight, after which it was centrifuged to (partially) remove the fine white particles, filtered over a 0.2 µm syringe filter and concentrated to dryness, yielding a dark red solid. The catalyst was dissolved in water and purified by dialysis (MW 3.500), affording 44 mg (2.86 µmol, 46%) of a dark red solid after concentration. ^1H NMR (300 MHz, CD_3OD): δ = 8.98–8.64 (m, 32H), 8.20–7.96 (m, 32H), 7.41–7.24 (m, 32H), 5.12–4.95 (m, 16H), 4.56–4.30 (m, 32H), 3.98–3.87 (m, 32H), 3.78–3.53 (m, 128H), 3.52–3.41 (m, 32H), 3.28–2.60 (m, 152H), 2.32–1.80 (m, 60H), 1.71–1.52 (m, 240H), 1.49–1.34 (m, 96H). ^{13}C NMR (125 MHz, CD_3OD): δ = 169.19, 168.35, 161.22, 158.26, 153.69, 116.59, 116.41, 112.55, 111.67, 89.95, 74.03, 71.80, 71.58, 71.48, 71.34, 70.65, 70.30, 69.92, 22.02, 21.88, 8.68. IR (ATR): 3364 (br), 2971, 2936, 2863, 1618, 1562, 1489, 1441, 1100 cm^{-1}. Mass spectrometry appeared unsuited for characterization, since no useful peaks could be detected with ESI–TOF or MALDI–TOF measurements.

DAB-G4-(Cp*Ir(bpy)SO_4)$_{32}$ (17): The method for the G3 analogue was followed with DAB-G4-(Cp*Ir(bpy)Cl_2)$_{32}$ (**15**) (90 mg, 2.99 µmol) and Ag_2SO_4 (29.87 mg, 95.8 µmol, 1 equiv) as starting materials and afforded 43 mg (1.39 µmol, 47%) of a dark red solid. ^1H NMR (300 MHz, CD_3OD): δ = 8.96–8.84 (m, 64H), 8.09–7.98 (m, 64H),

7.36–7.27 (m, 64H), 5.08–4.96 (m, 32H), 4.54–4.25 (m, 64H), 3.97–3.86 (m, 64H), 3.77–3.58 (m, 256H), 3.50–3.43 (m, 64H), 3.28–2.92 (m, 280H), 2.40–1.76 (m, 180H), 1.69–1.65 (m, 480H), 1.47–1.41 (m, 196H). ^{13}C NMR (125 MHz, CD_3OD): δ = 169.07, 168.34, 161.17, 158.24, 153.69, 116.82, 116.66, 112.51, 111.55, 89.96, 74.03, 71.82, 71.64, 71.59, 71.50, 71.35, 70.83, 70.26, 22.05, 21.87, 8.70. IR (ATR): 3386, 2975, 2923, 2872, 1614, 1562, 1484, 1454, 1100 cm^{-1}. Mass spectrometry appeared unsuited for characterization, since no useful peaks could be detected with ESI–TOF or MALDI–TOF measurements.

***N*-benzyl-L-valine (19):** Synthesis for the HPLC analysis was performed according to a literature description [5]. Benzaldehyde (2.04 mL, 20 mmol, 1 equiv) was added to a solution of L-valine (2.34 g, 20 mmol) in 10 mL 2 M NaOH. The mixture was stirred for 90 minutes, after which $NaBH_4$ (0.227 g, 6.00 mmol, 0.3 equiv) was gradually added at 0 °C. The mixture was allowed to warm to room temperature and another portion of benzaldehyde (2.04 mL, 20 mmol, 1 equiv) was added after 90 minutes. The mixture was cooled to 0 °C and $NaBH_4$ (0.227 g, 6.00 mmol, 0.3 equiv) was portionwise added after 45 minutes. The mixture was stirred for 2 h at room temperature, diluted with 10 mL water and washed twice with DCM (10 mL). The aqueous phase was acidified until a neutral pH was achieved. The white precipitate was isolated by filtration and washed with water and acetone, yielding 3.65 g (17.6 mmol, 88%) of a white powder. ^{1}H NMR (400 MHz, D_2O + NaOH): δ = 7.58–6.88 (m, 5H), 3.69–3.59 (m, 1H), 3.46–3.59 (m, 1H), 2.93–2.48 (m, 1H), 1.96–1.37 (m, 1H), 0.99–0.40 (m, 6H). ^{13}C NMR (75 MHz, D_2O) δ = 181.20, 138.47, 128.14, 128.05, 126.73, 68.32, 50.94, 30.16, 18.26, 17.92. IR (ATR): 3420 (br), 3040, 2962, 2872, 1605, 1571, 1437, 1406, 1320, 1221 cm^{-1}.

References

1. McFarland, J. M.; Francis, M. B. *J. Am. Chem. Soc.* **2005,** *127,* 13490–13491. doi:10.1021/ja054686c
2. Hong, Y.-R.; Gorman, C. B. *J. Org. Chem.* **2003,** *68,* 9019–9025. doi:10.1021/jo0351116
3. Kolodziejczyk, A. M.; Manning, M. *J. Org. Chem.* **1981,** *46,* 1944–1946. doi:10.1021/jo00322a049
4. Pope, B. M.; Yamamoto, Y.; Stanley Tarbell, D. *Org. Synth.* **1977,** *57,* 45–49.
5. Quitt, P.; Hellerbach, J.; Vogler, K. *Helv. Chim. Acta* **1963,** *46,* 327–333. doi:10.1002/hlca.19630460133
