# Supplementary material for: Aqueous reductive amination using a dendritic metal catalyst in a dialysis bag
Source: Beilstein J Org Chem. 2013 May 17;9:960–5. doi: 10.3762/bjoc.9.110 (PMC3678615; doi:10.3762/bjoc.9.110)
Supplement: File 2 — Spectra of compounds. [file Beilstein_J_Org_Chem-09-960-s002.pdf]

Supporting Information File 2

for

Aqueous reductive amination using a dendritic metal catalyst in a dialysis bag

Jorgen S. Willemsen, Jan C. M. van Hest* and Floris P. J. T. Rutjes*

Address: Radboud University Nijmegen, Institute for Molecules and Materials, Heyendaalseweg 135, 6525 AJ Nijmegen, The Netherlands

Email: Jan C. M. van Hest - J.vanHest@science.ru.nl, Floris P. J. T. Rutjes - F.Rutjes@science.ru.nl

* Corresponding author

Spectra of compounds

2 ^{1}H NMR

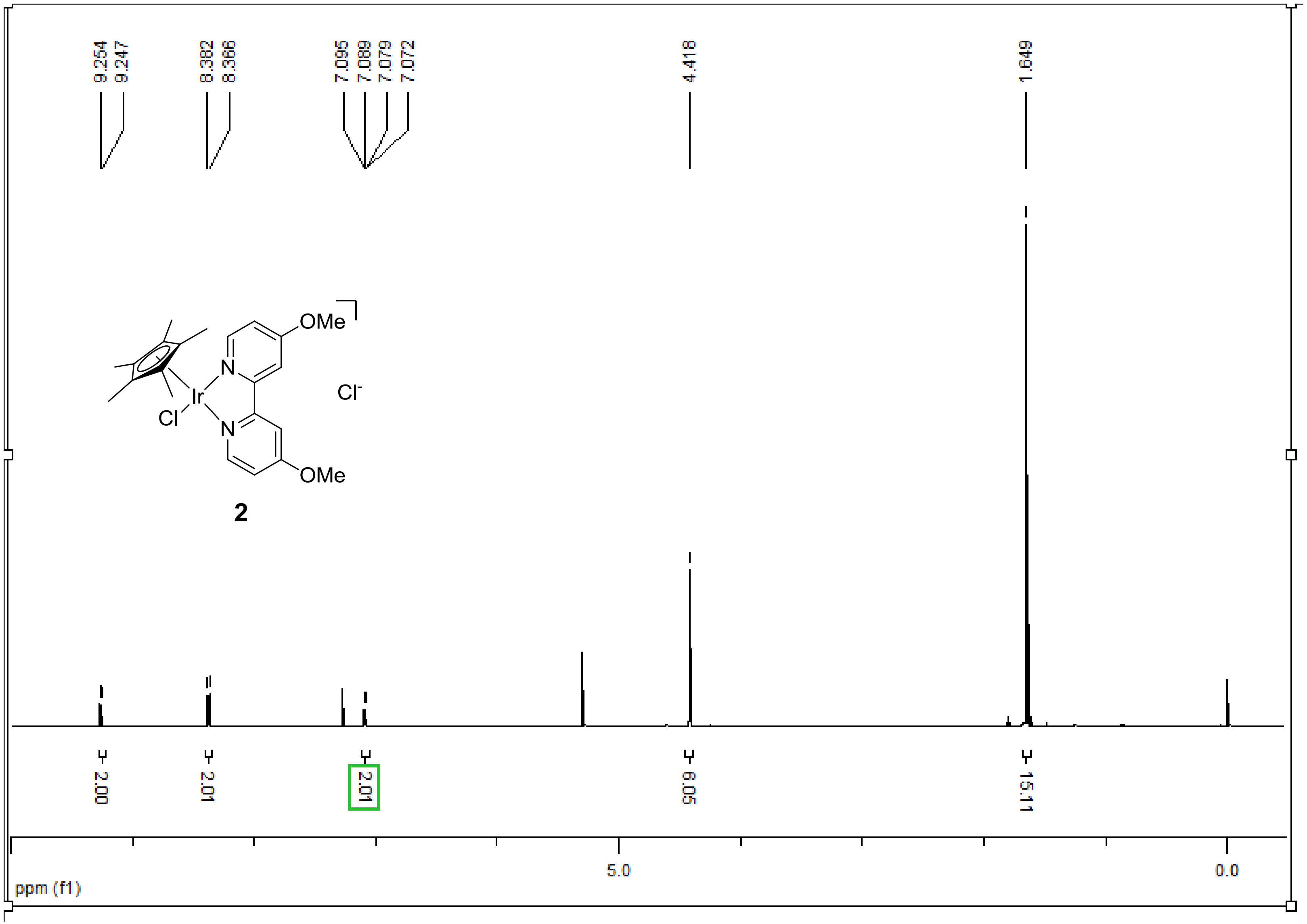

9.254
9.247
8.382
8.366
7.095
7.089
7.079
7.072
4.418
1.649
OMe
N
Ir
Cl
N
OMe
Cl-
2
2.00
2.01
2.01
6.05
15.11
5.0
0.0
ppm (f1)

2 ^{13}C NMR

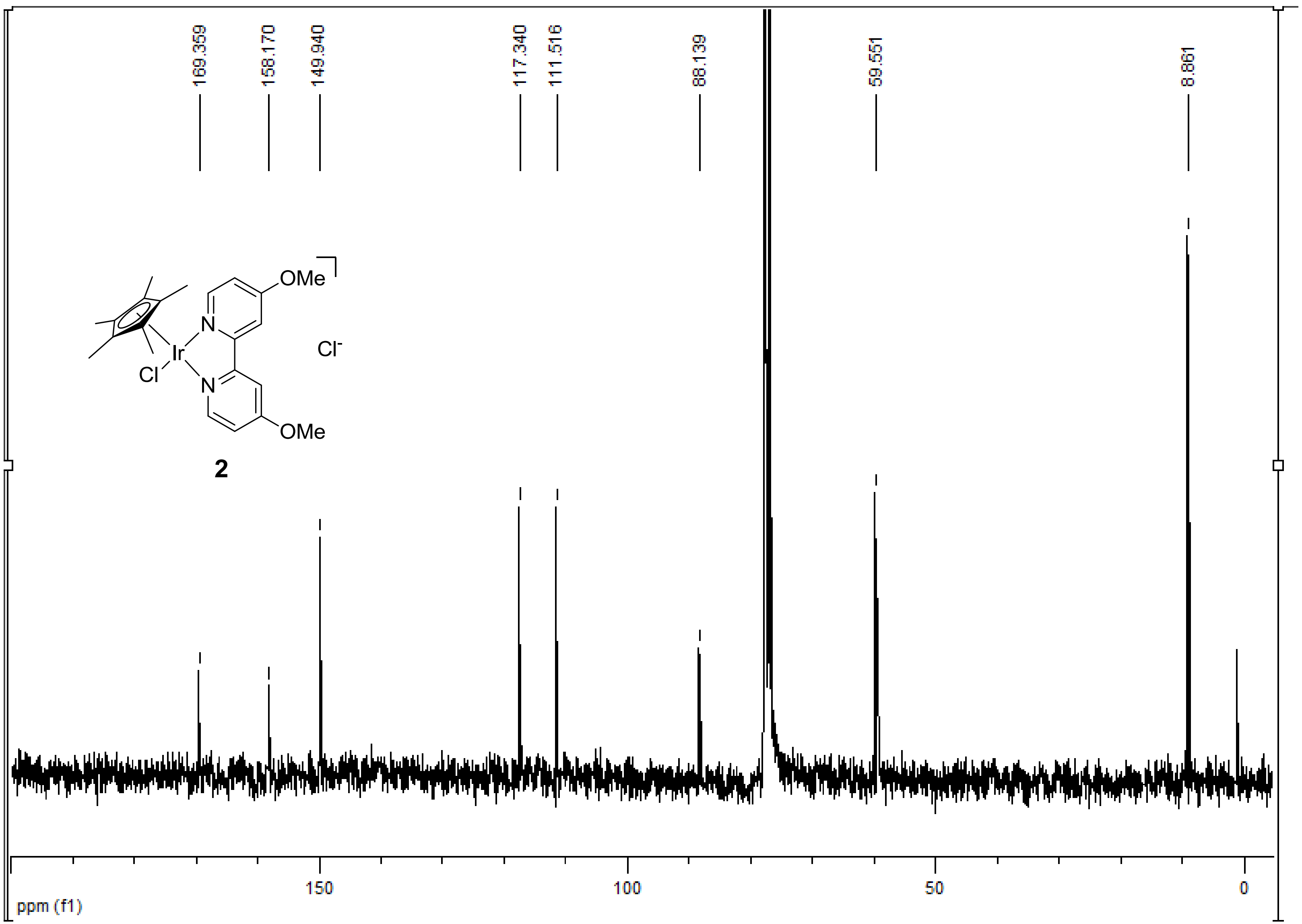

169.359
158.170
149.940
117.340
111.516
88.139
59.551
8.861
OMe
N
Ir
Cl
N
OMe
Cl-
2
150
100
50
0
ppm (f1)

3 ^{1}H NMR

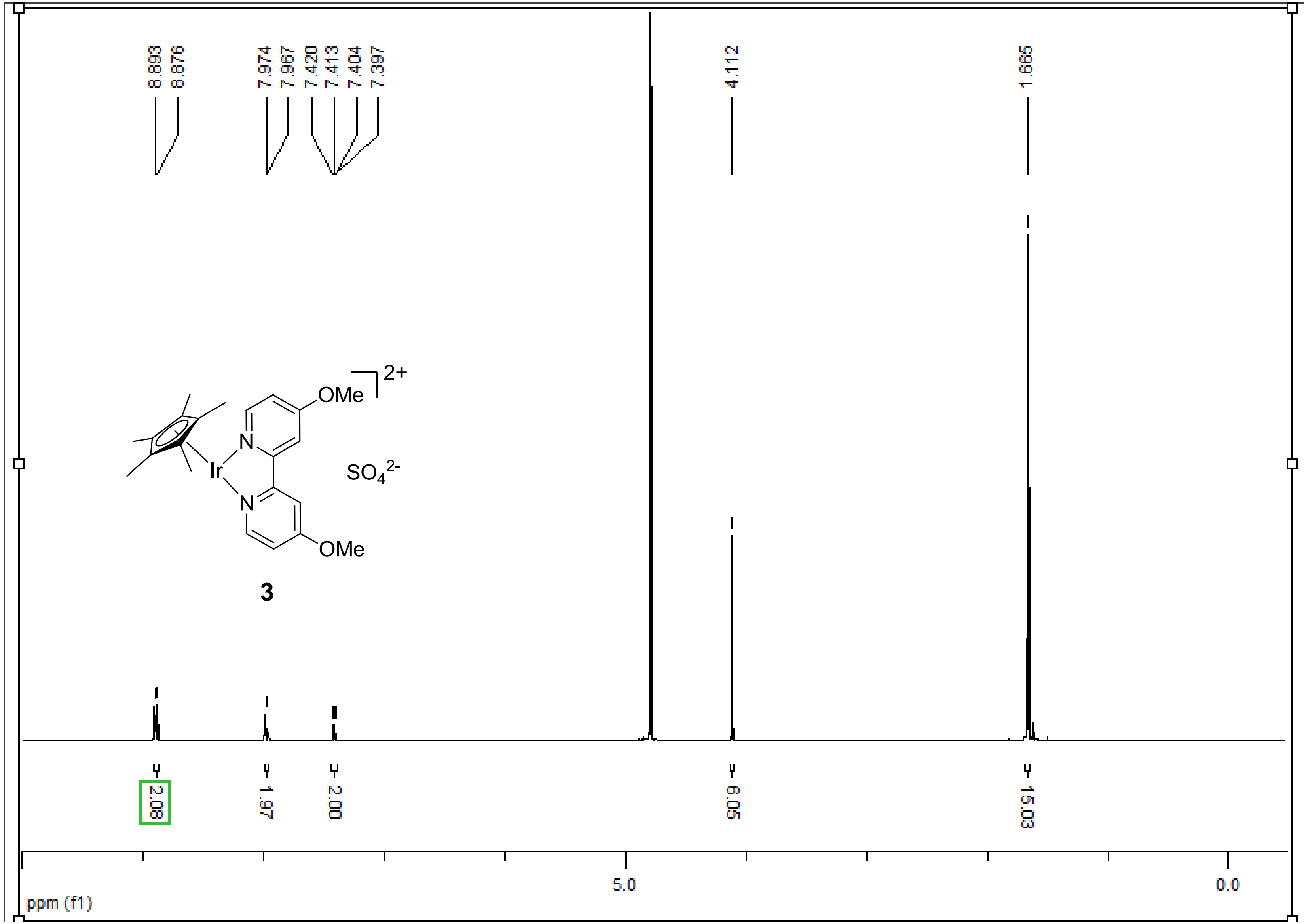

8.893
8.876
7.974
7.967
7.420
7.413
7.404
7.397
4.112
1.665
OMe
2+
N
Ir
N
SO$_4^{2-}$
OMe
3
2.08
1.97
2.00
6.05
15.03
5.0
0.0
ppm (f1)

3 (^{13}C NMR)

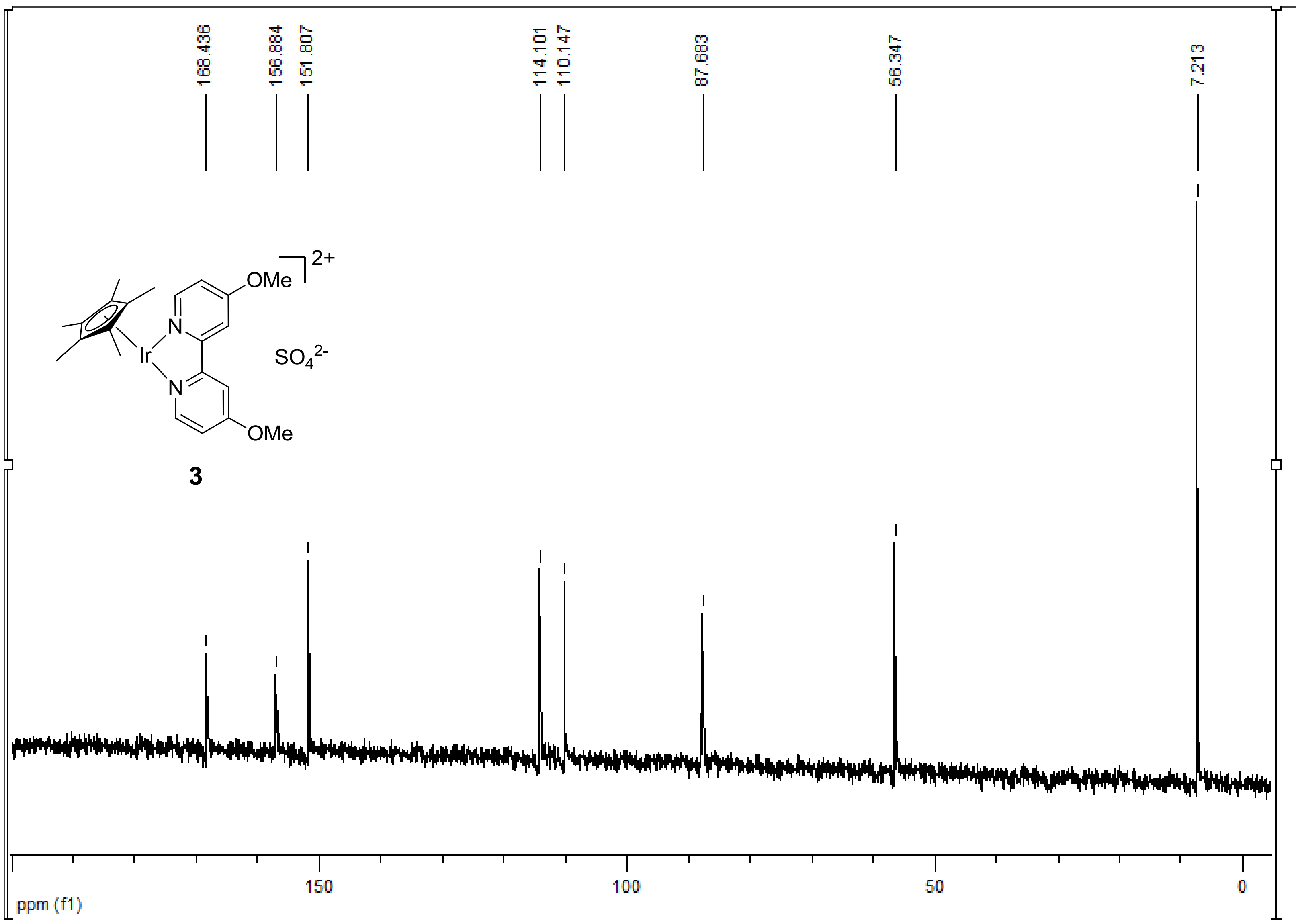
168.436
156.884
151.807
114.101
110.147
87.683
56.347
7.213
OMe
2+
N
Ir
N
SO4 2-
OMe
3
150
100
50
0
ppm (f1)

4 ^{1}H NMR

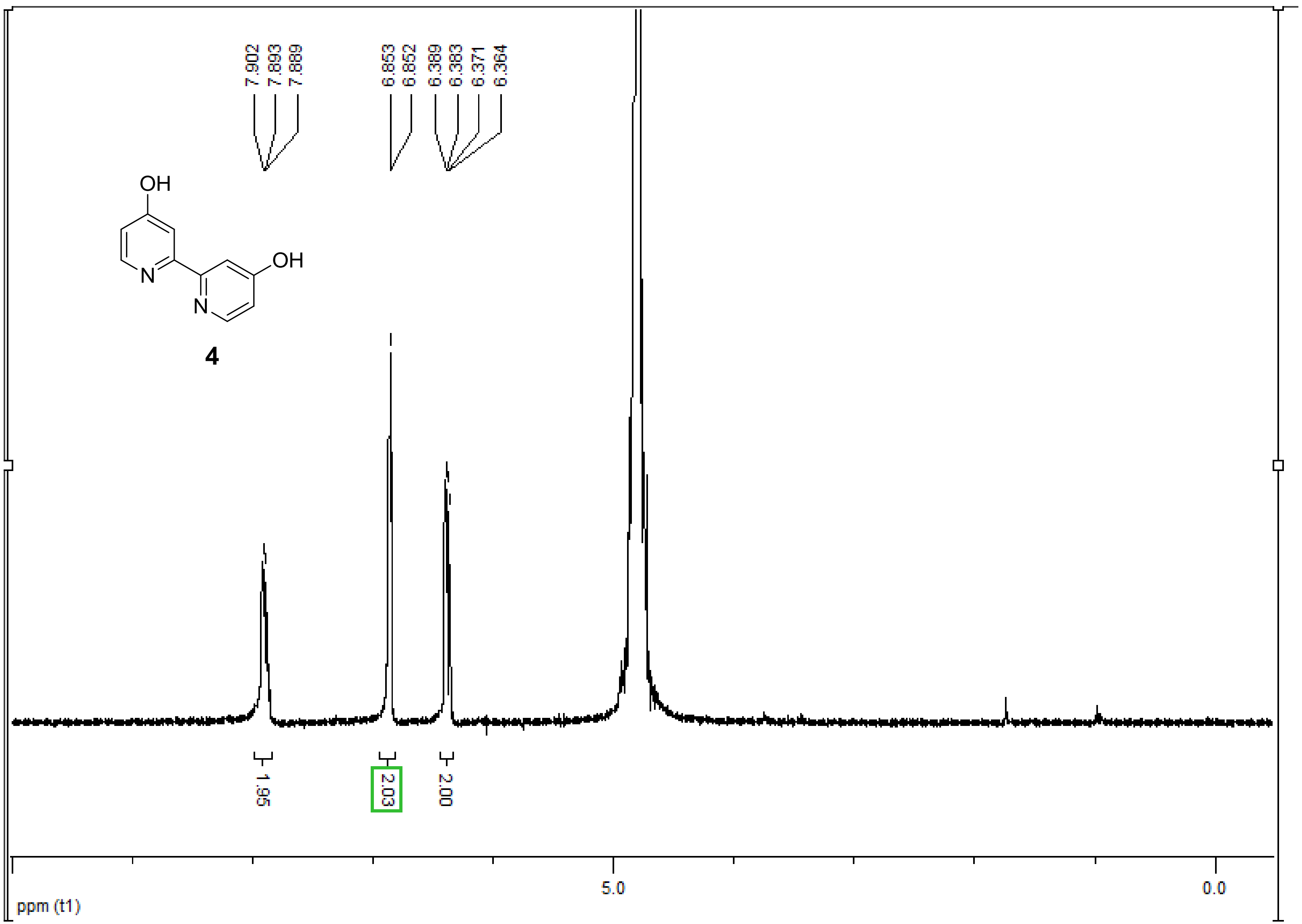
7.902
7.893
7.889
6.853
6.852
6.389
6.383
6.371
6.364
OH
OH
N
N
4
1.95
2.03
2.00
5.0
0.0
ppm (t1)

4 ^{13}C NMR

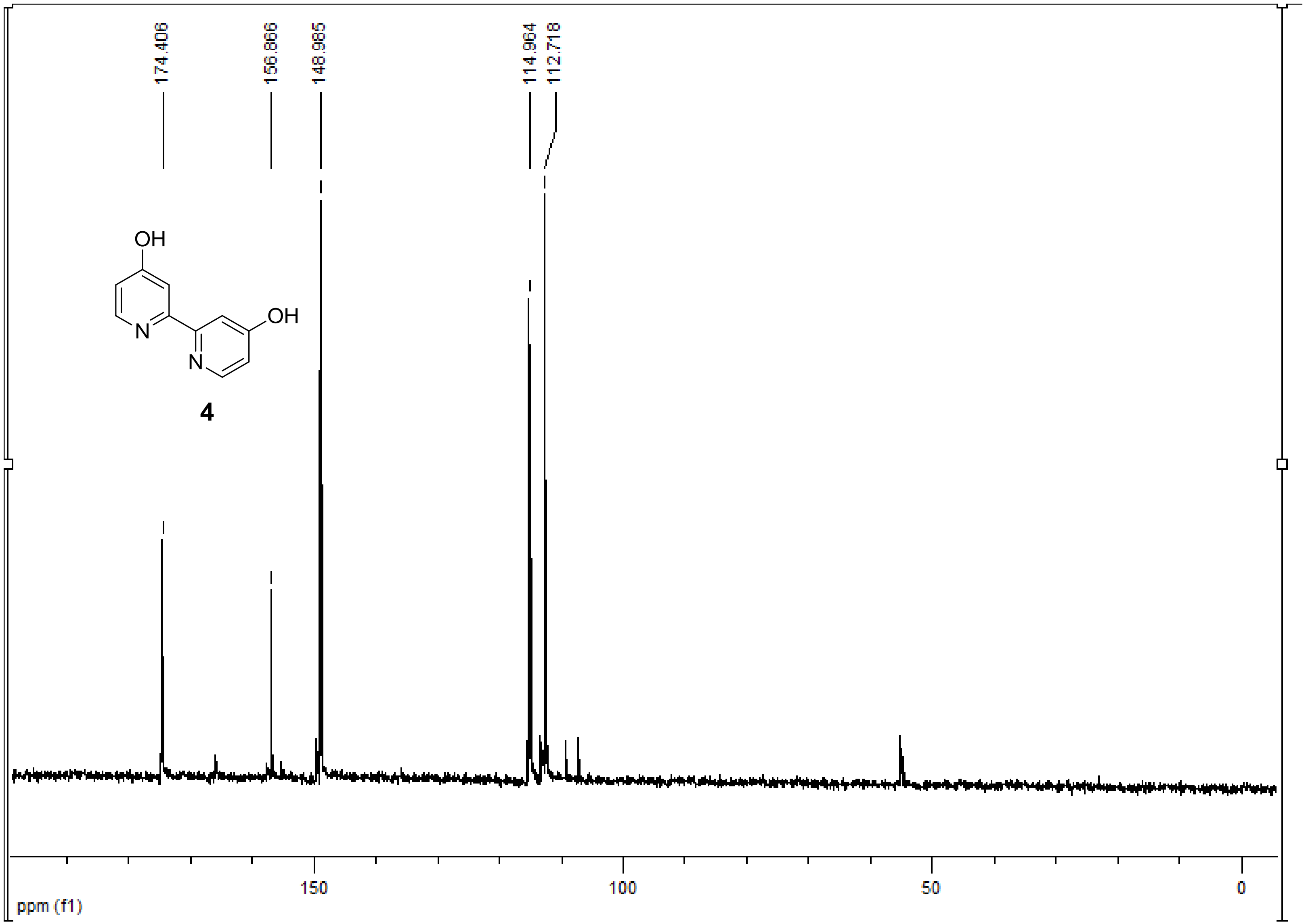
174.406
156.866
148.985
114.964
112.718
OH
OH
N
N
4
150
100
50
0
ppm (f1)

5 ^{1}H NMR

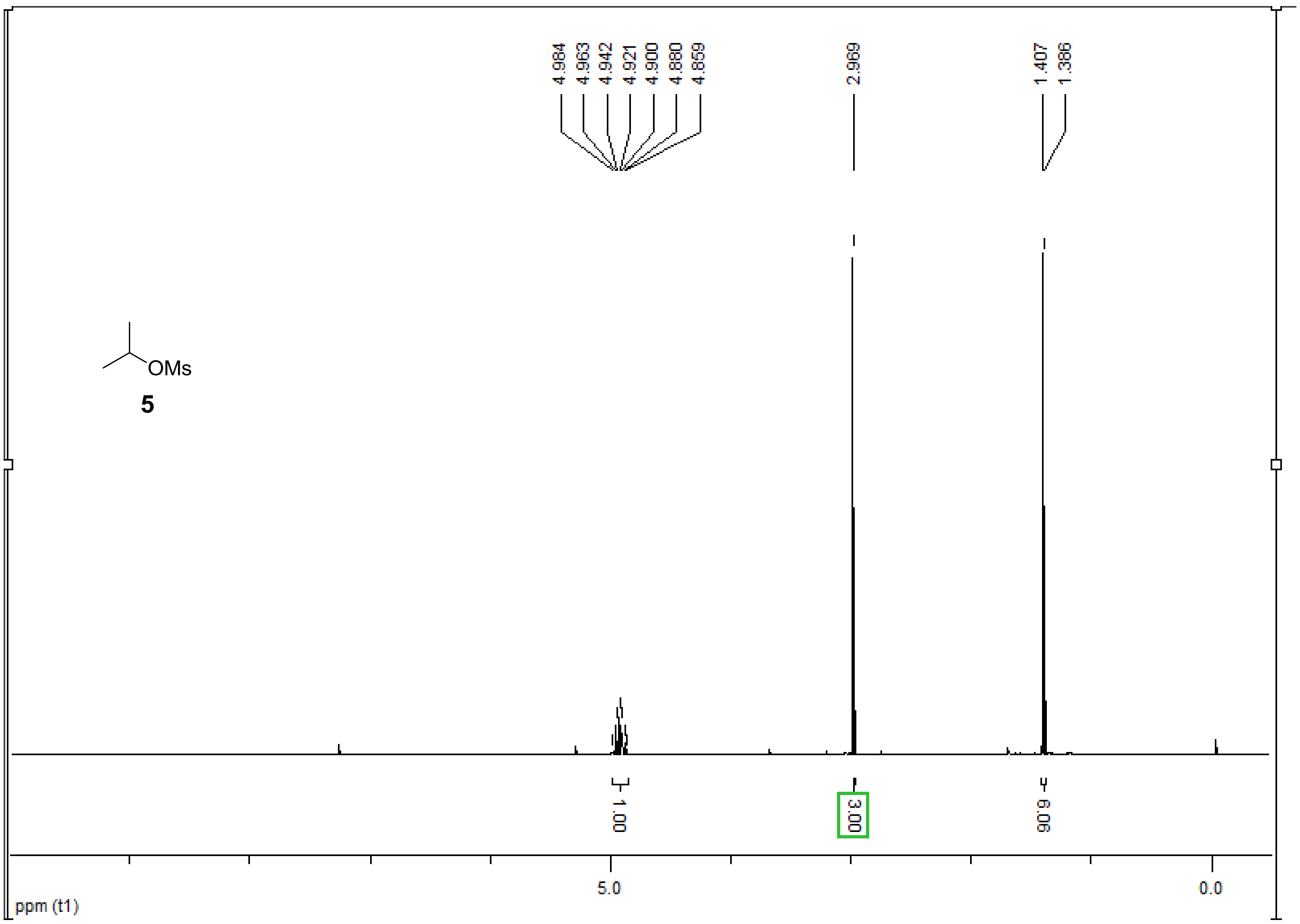

4.984
4.963
4.942
4.921
4.900
4.880
4.859
2.969
1.407
1.386
OMs
5
1.00
3.00
6.06
5.0
0.0
ppm (t1)

5 ^{13}C NMR

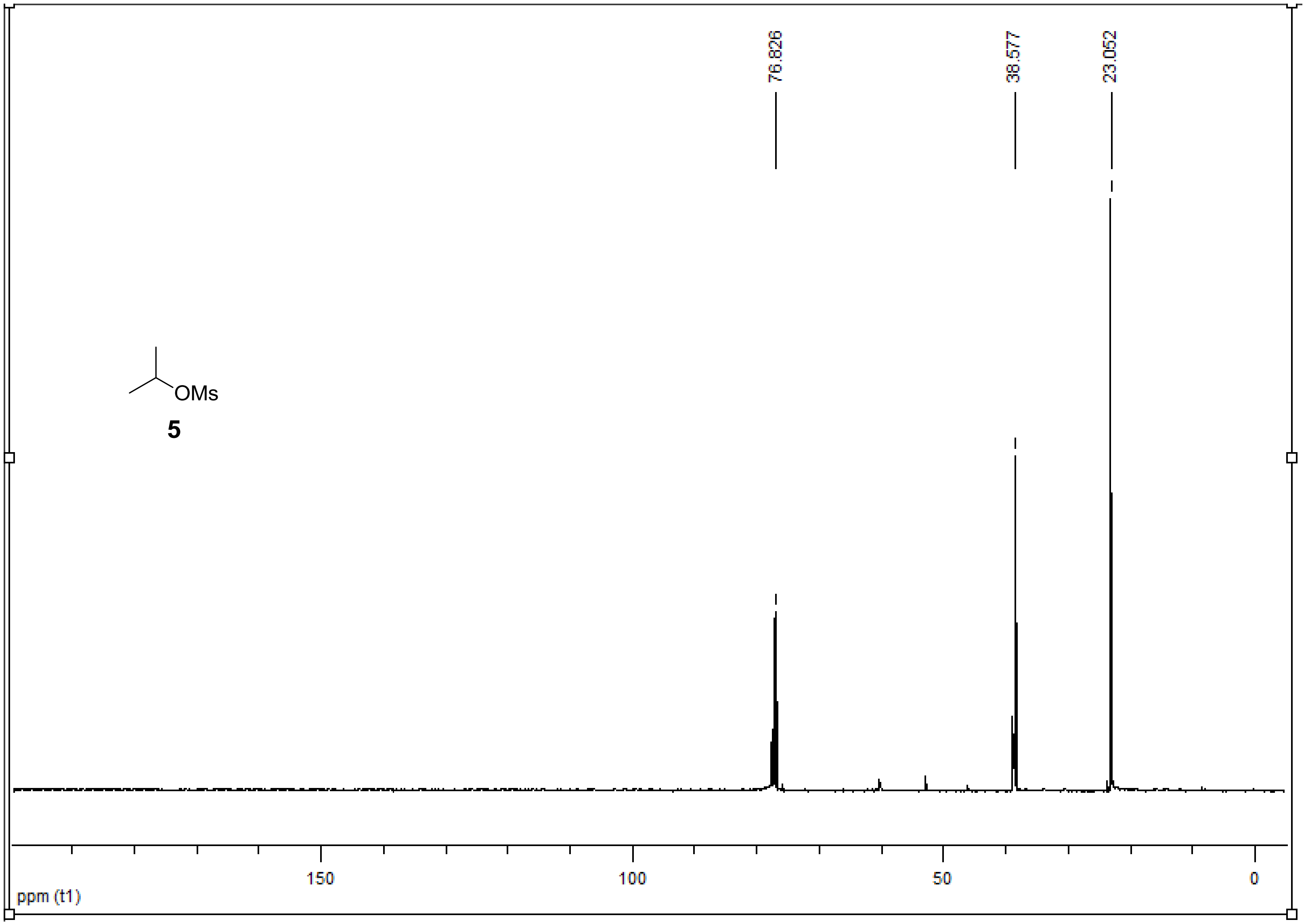

6a ^{1}H NMR

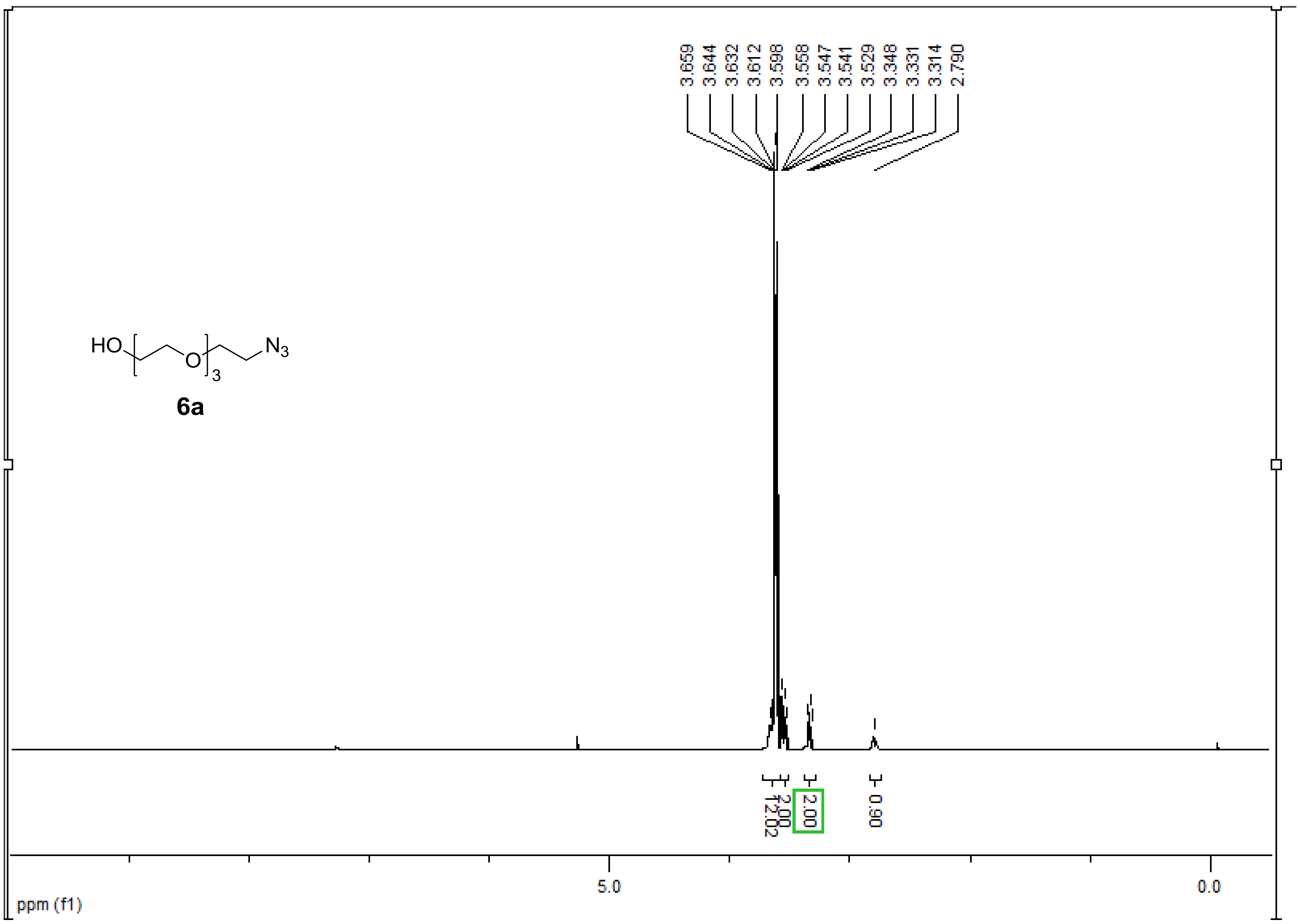

3.659
3.644
3.632
3.612
3.598
3.558
3.547
3.541
3.529
3.348
3.331
3.314
2.790
HO
O
3
N3
6a
12.02
2.00
2.00
0.90
5.0
0.0
ppm (f1)

6a ^{13}C NMR

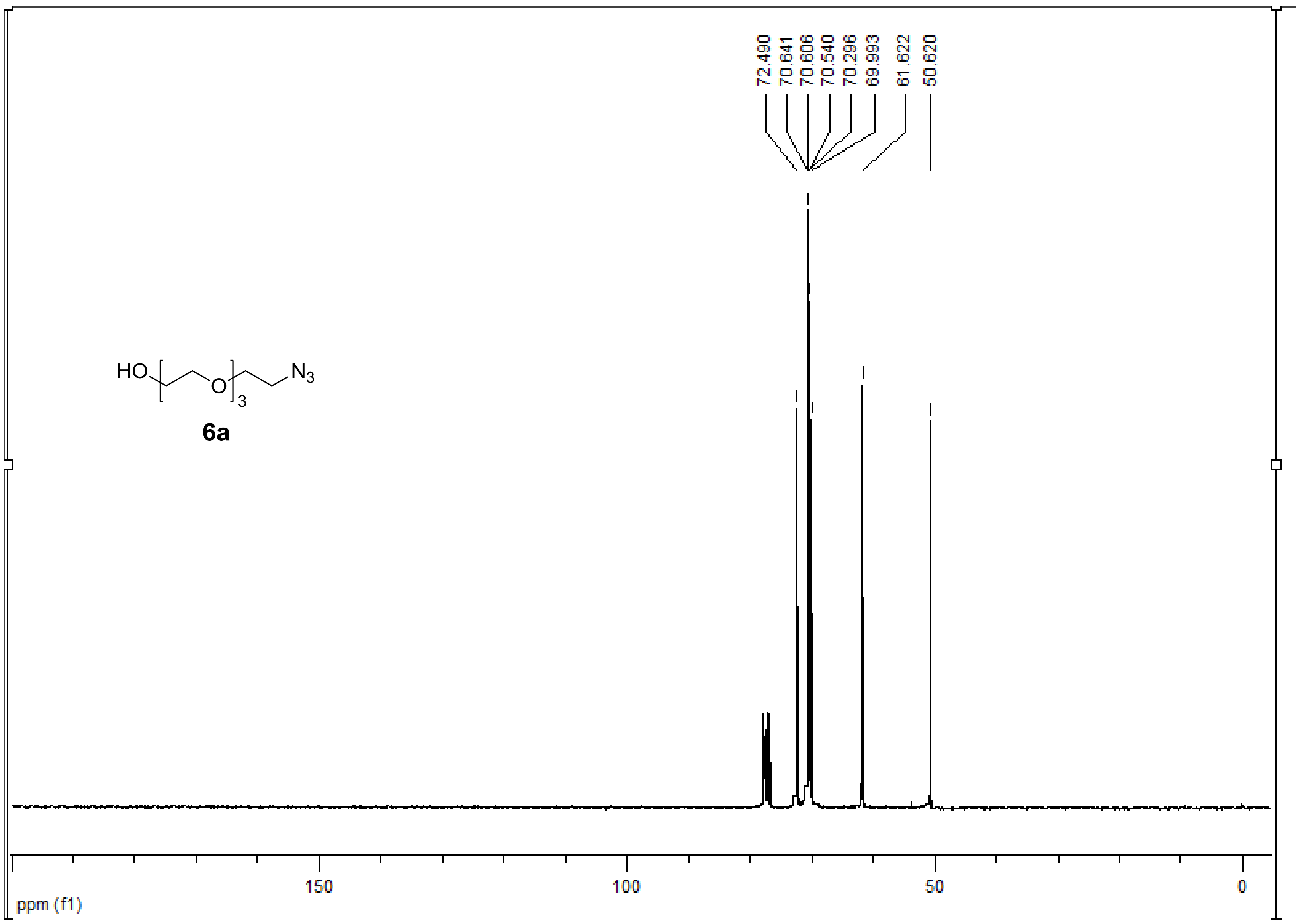
72.490
70.641
70.606
70.540
70.296
69.993
61.622
50.620
HO
O
3
N3
6a
150
100
50
0
ppm (f1)

6 ^{1}H NMR

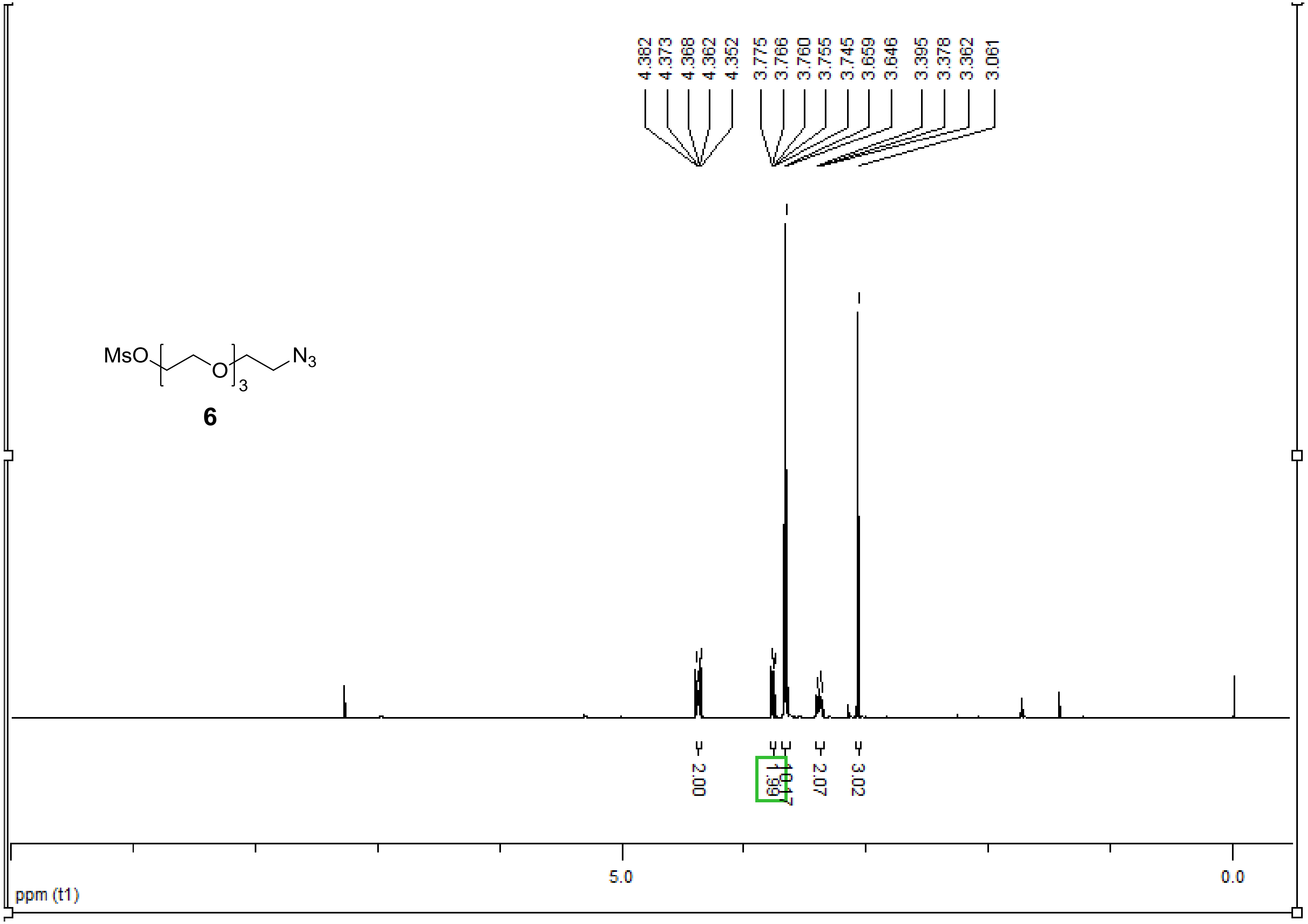
4.382
4.373
4.368
4.362
4.352
3.775
3.766
3.760
3.755
3.745
3.659
3.646
3.395
3.378
3.362
3.061
MsO
O
3
N3
6
2.00
1.99
10.17
2.07
3.02
5.0
0.0
ppm (t1)

6 ^{13}C NMR

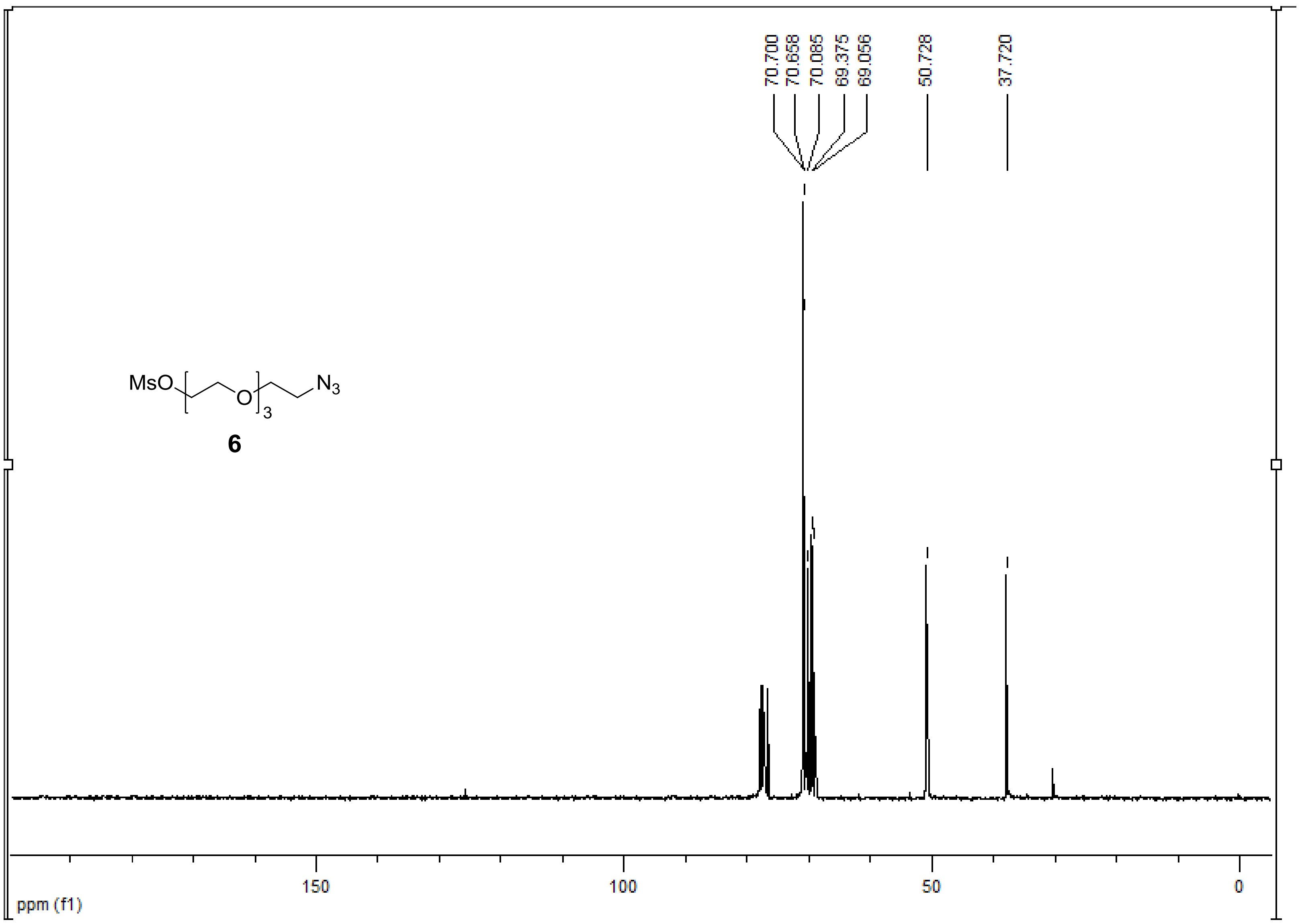

70.700
70.658
70.085
69.375
69.056
50.728
37.720
MsO
O
3
N3
6
150
100
50
0
ppm (f1)

7 ^{1}H NMR

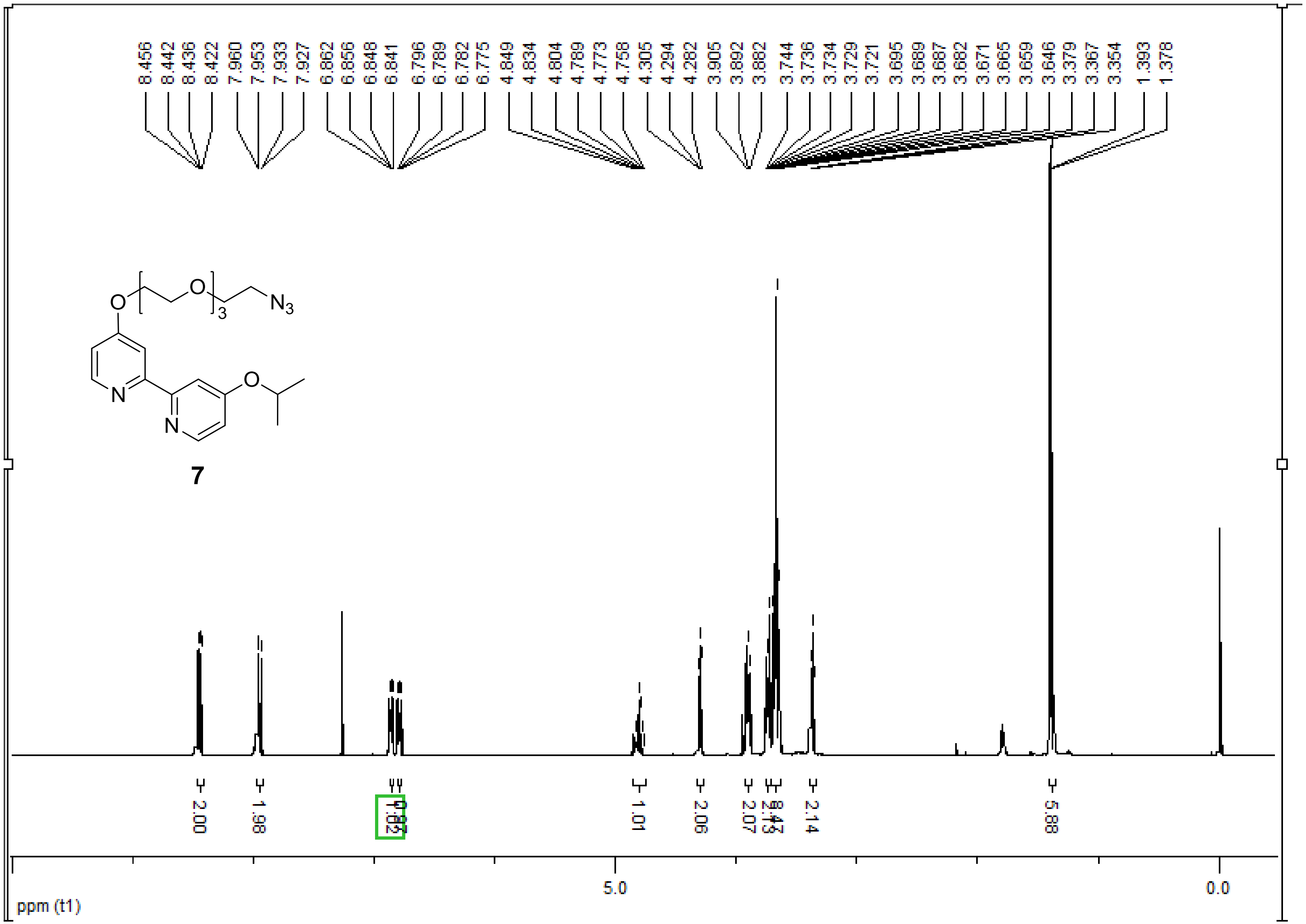
8.456
8.442
8.436
8.422
7.960
7.953
7.933
7.927
6.862
6.856
6.848
6.841
6.796
6.789
6.782
6.775
4.849
4.834
4.804
4.789
4.773
4.758
4.305
4.294
4.282
3.905
3.892
3.882
3.744
3.736
3.734
3.729
3.721
3.695
3.689
3.687
3.682
3.671
3.665
3.659
3.646
3.379
3.367
3.354
1.393
1.378
7
2.00
1.98
1.01
2.06
2.07
2.14
5.88
5.0
0.0
ppm (t1)

7 ^{13}C NMR

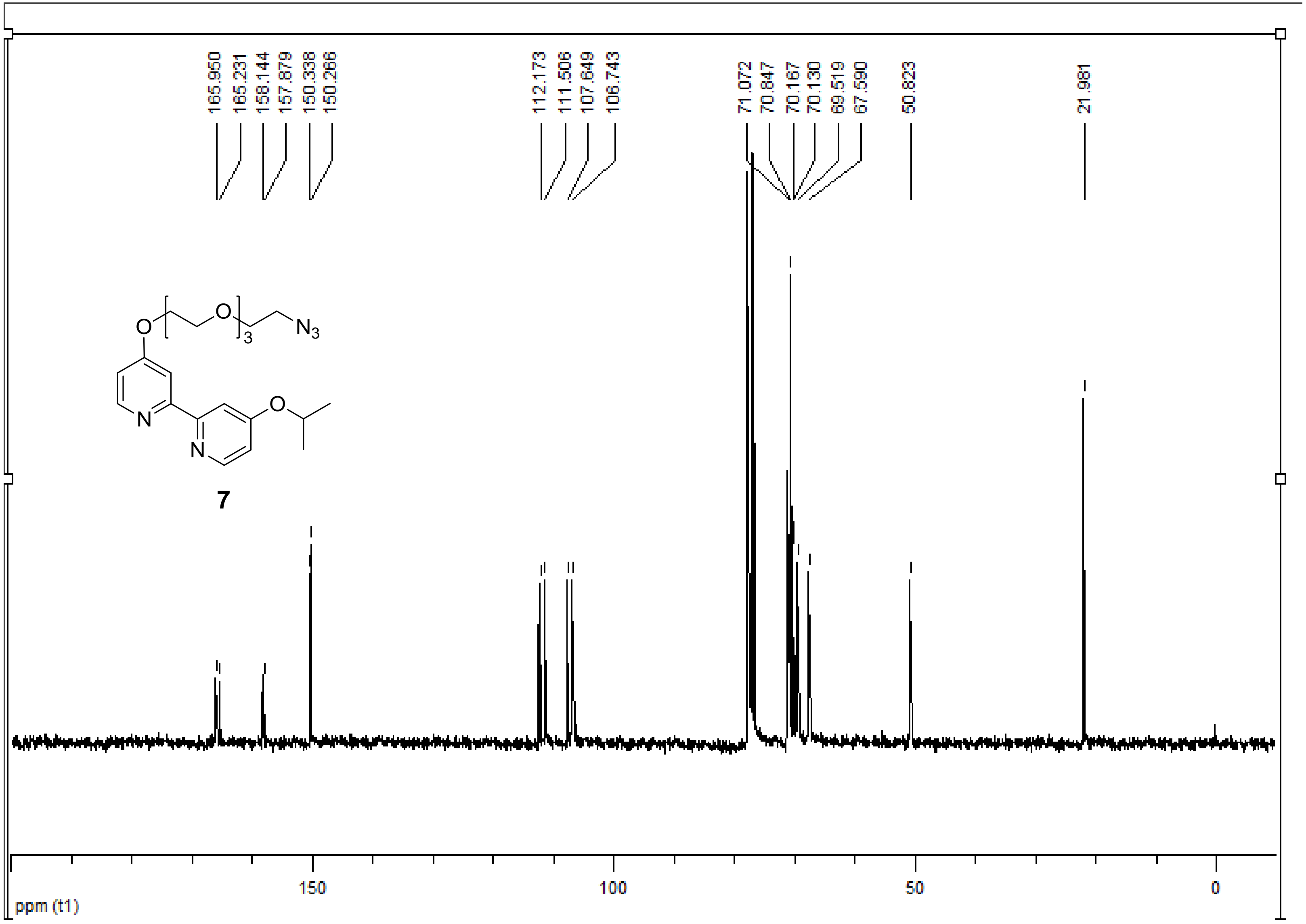
165.950
165.231
158.144
157.879
150.338
150.266
112.173
111.506
107.649
106.743
71.072
70.847
70.167
70.130
69.519
67.590
50.823
21.981
O
O
3
N3
N
N
O
7
150
100
50
0
ppm (t1)

8 ^{1}H NMR

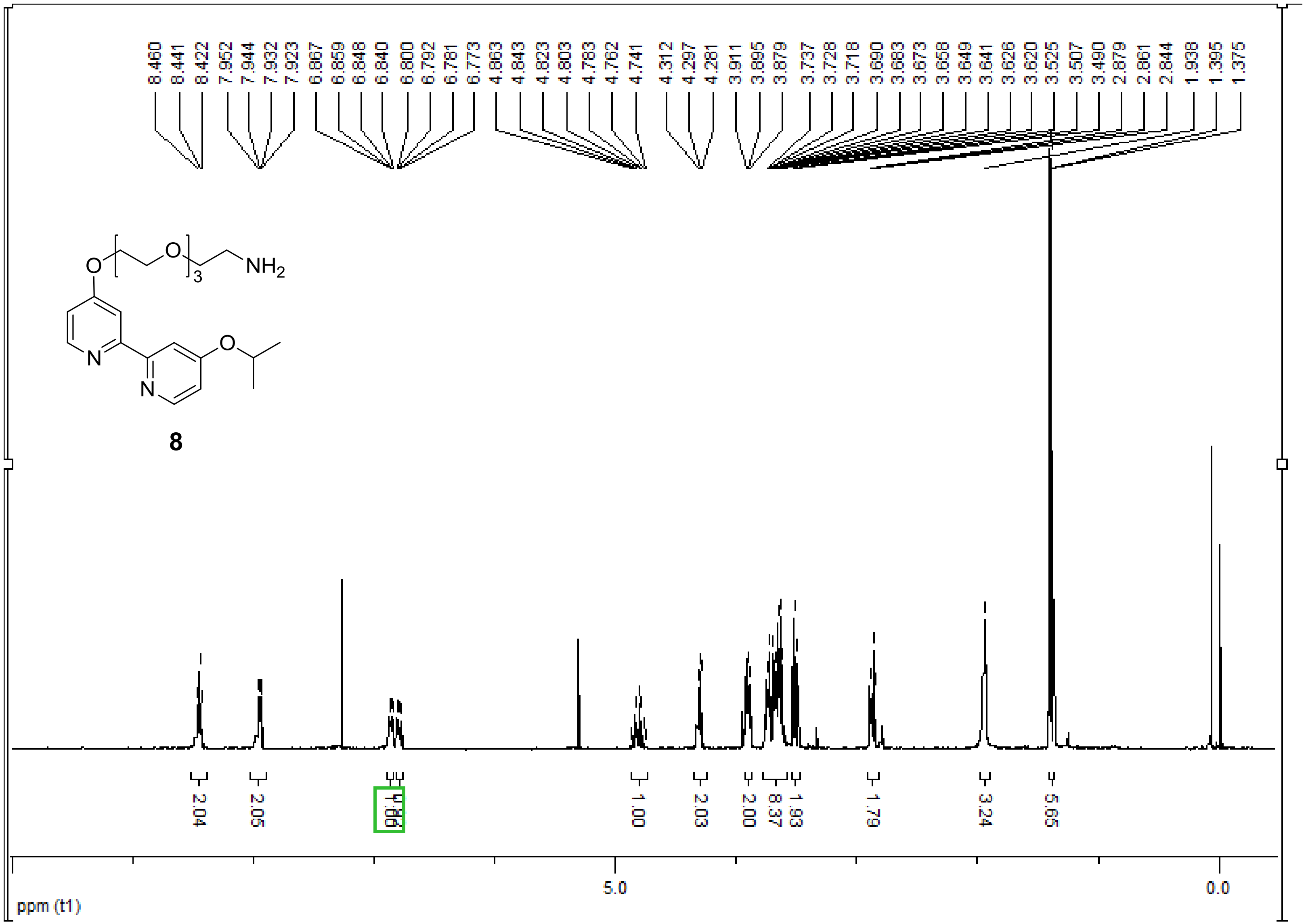

8

8 ^{13}C NMR

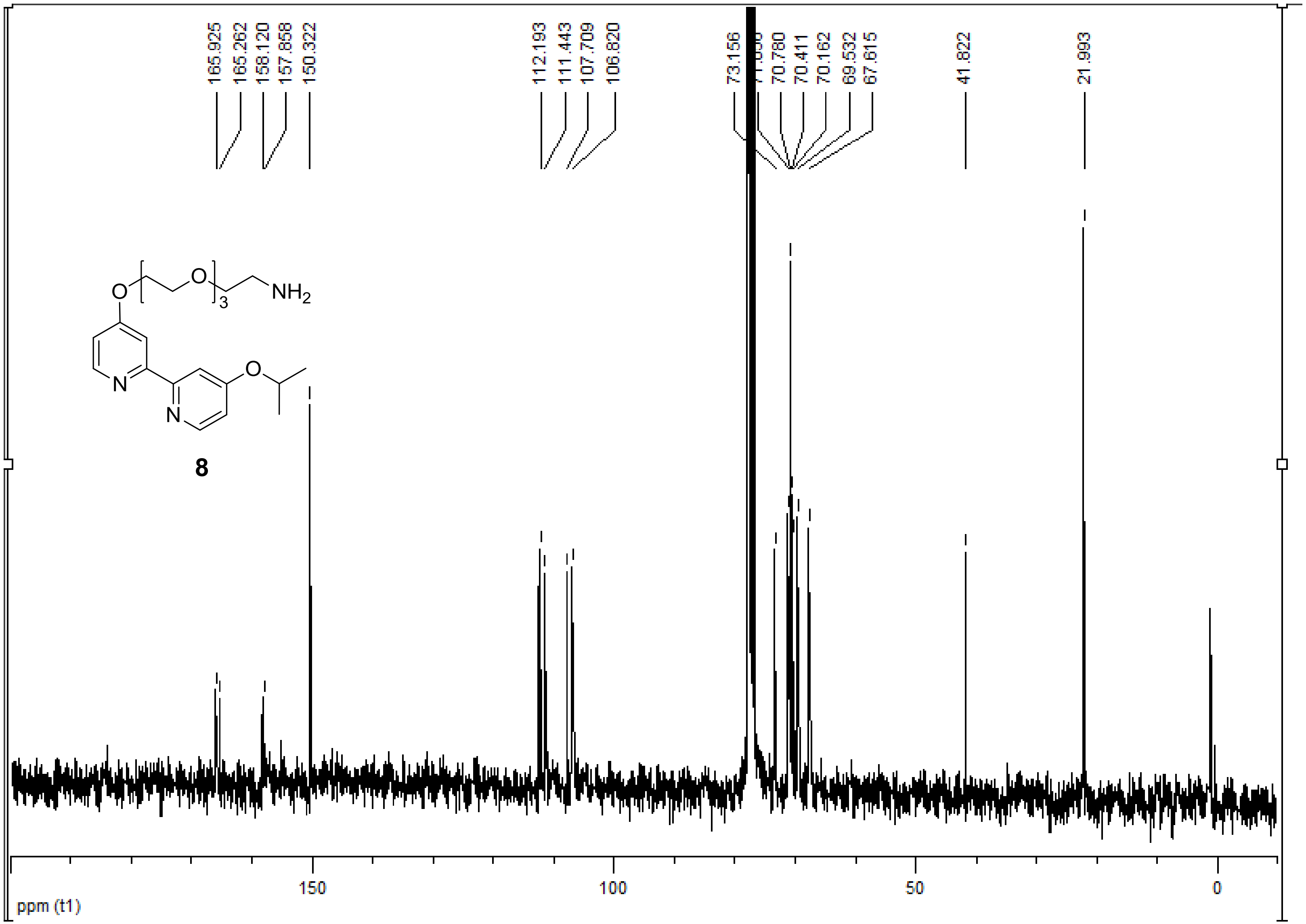
165.925
165.262
158.120
157.858
150.322
112.193
111.443
107.709
106.820
73.156
70.780
70.411
70.162
69.532
67.615
41.822
21.993
O
O
3
NH2
O
N
N
8
150
100
50
0
ppm (t1)

11 ^{1}H NMR

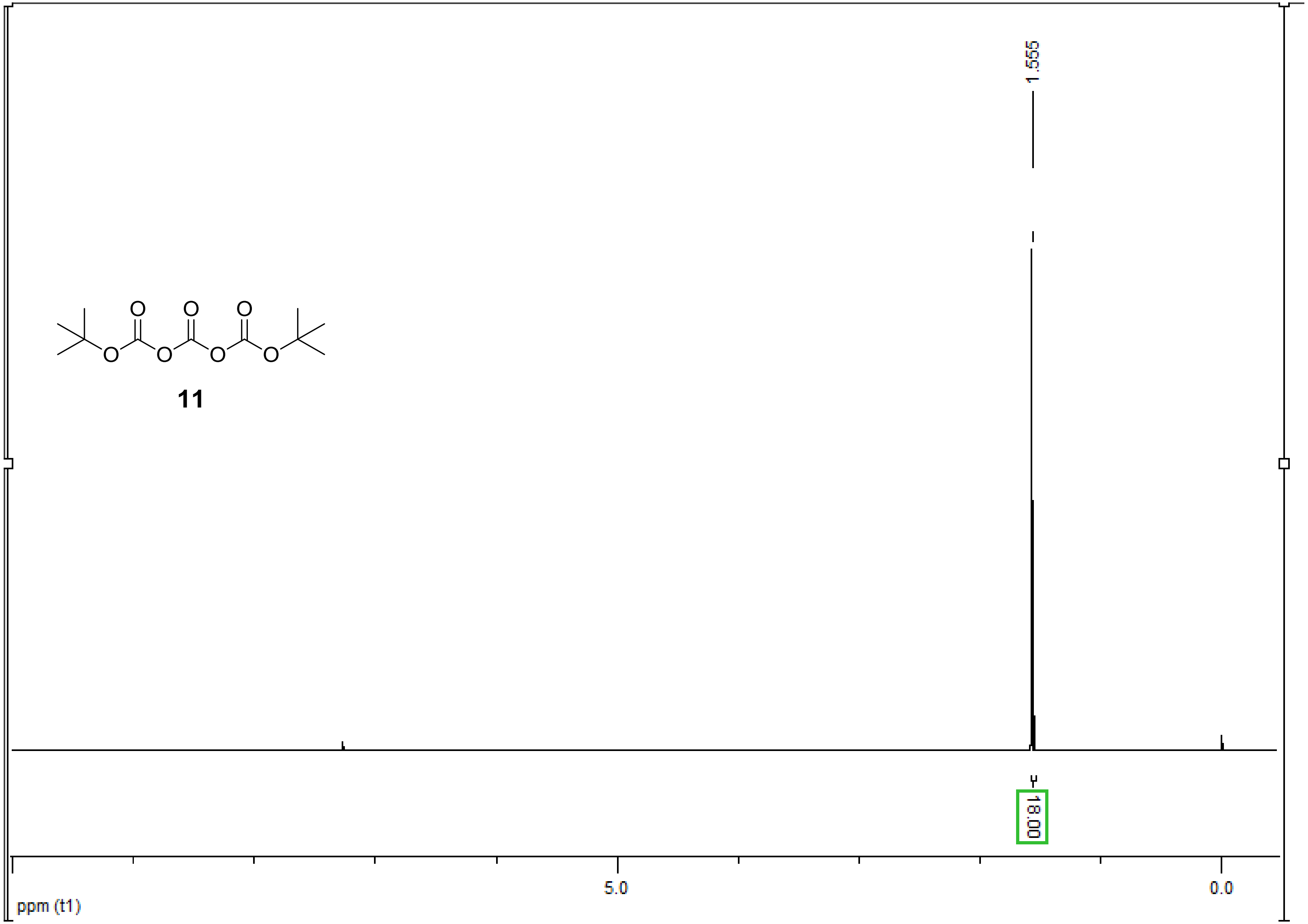

11 ^{13}C NMR

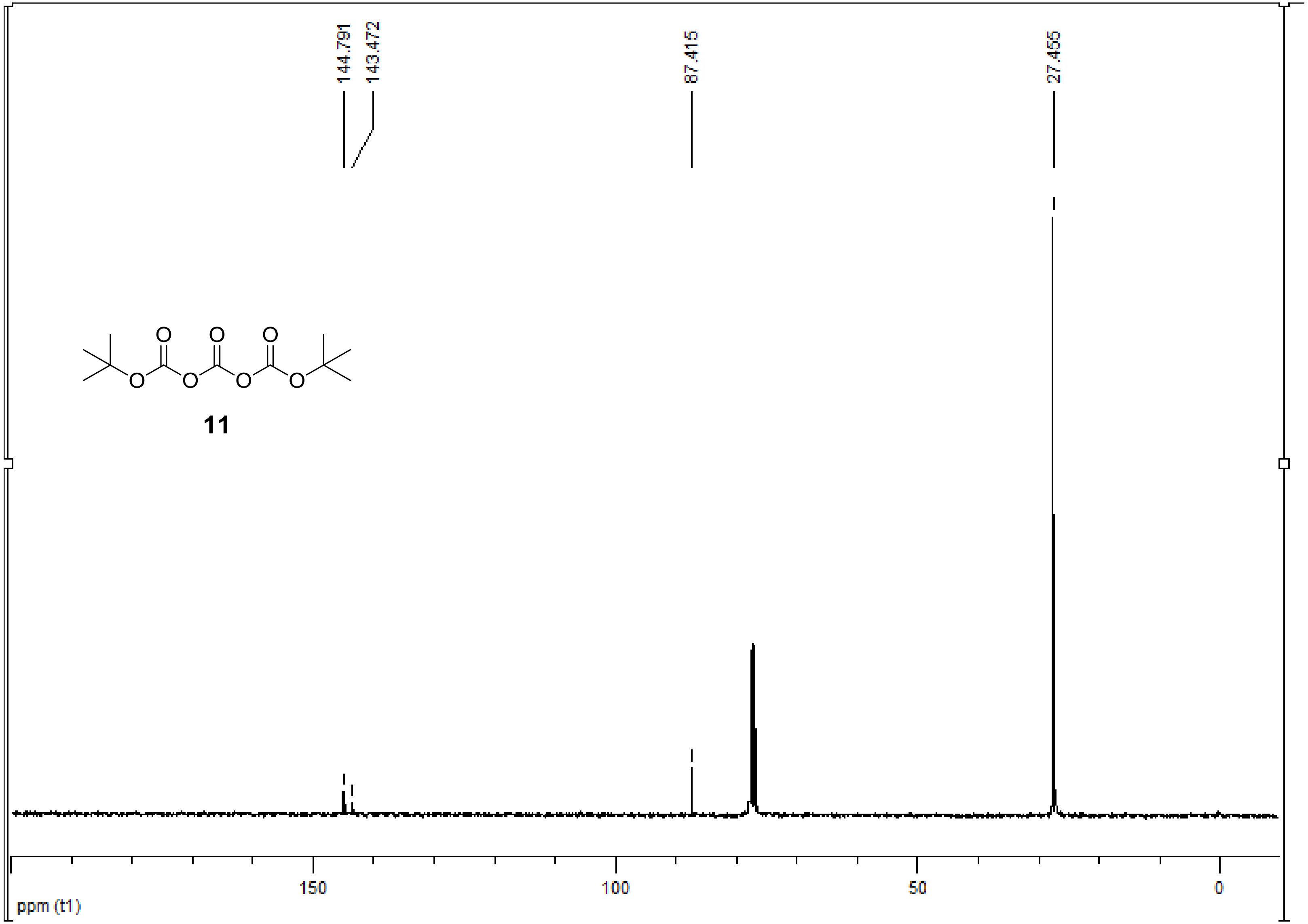

12 ^{1}H NMR

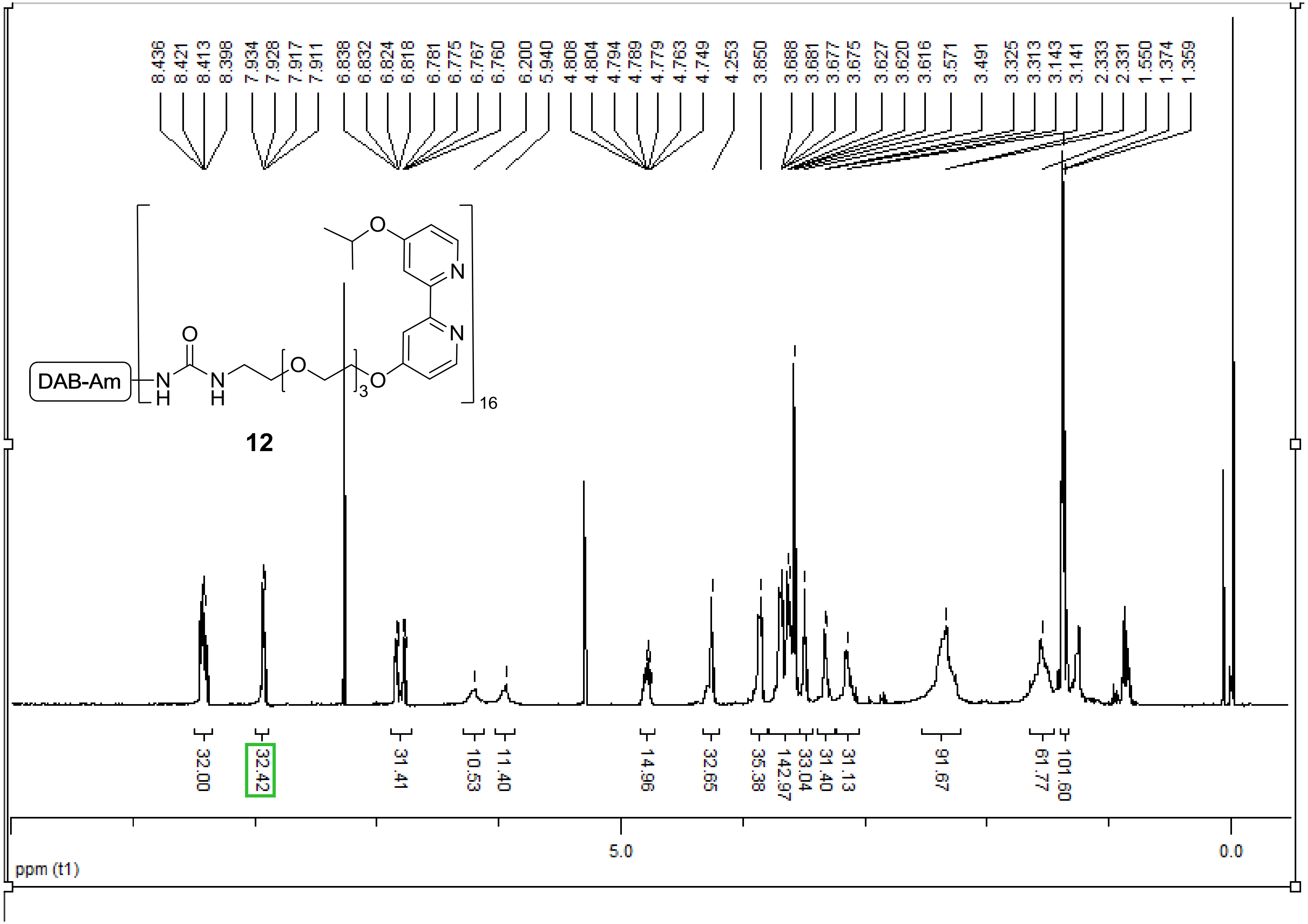
8.436
8.421
8.413
8.398
7.934
7.928
7.917
7.911
6.838
6.832
6.824
6.818
6.781
6.775
6.767
6.760
6.200
5.940
4.808
4.804
4.794
4.789
4.779
4.763
4.749
4.253
3.850
3.688
3.681
3.677
3.675
3.627
3.620
3.616
3.571
3.491
3.325
3.313
3.143
3.141
2.333
2.331
1.550
1.374
1.359
DAB-Am
12
32.00
32.42
31.41
10.53
11.40
14.96
32.65
35.38
142.97
33.04
31.40
31.13
91.67
61.77
101.60
5.0
0.0
ppm (t1)

12 ^{13}C NMR

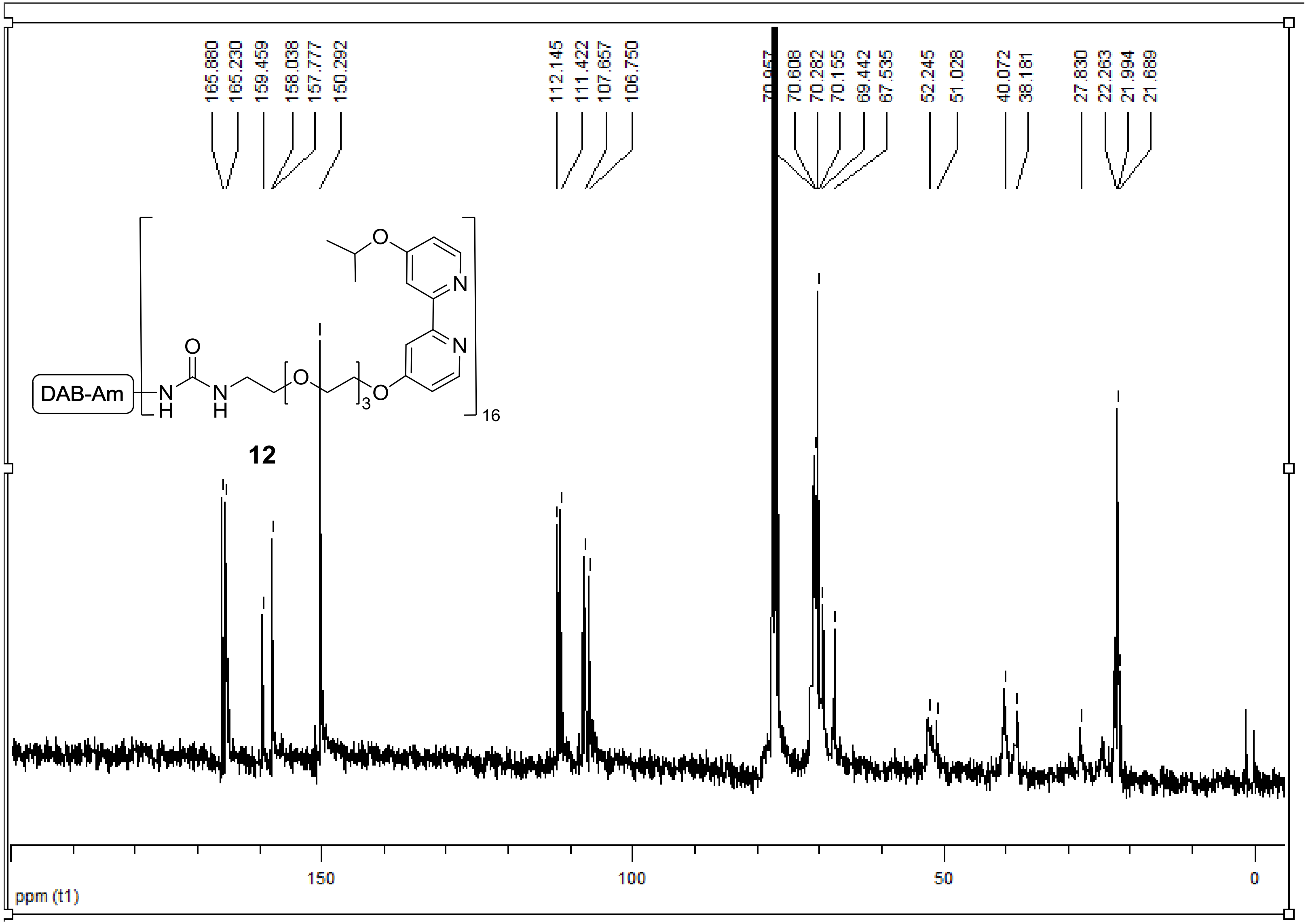

165.880
165.230
159.459
158.038
157.777
150.292
112.145
111.422
107.657
106.750
70.608
70.282
70.155
69.442
67.535
52.245
51.028
40.072
38.181
27.830
22.263
21.994
21.689
DAB-Am
N
H
O
N
H
O
3
O
N
N
O
16
12
150
100
50
0
ppm (t1)

13 ^{1}H NMR

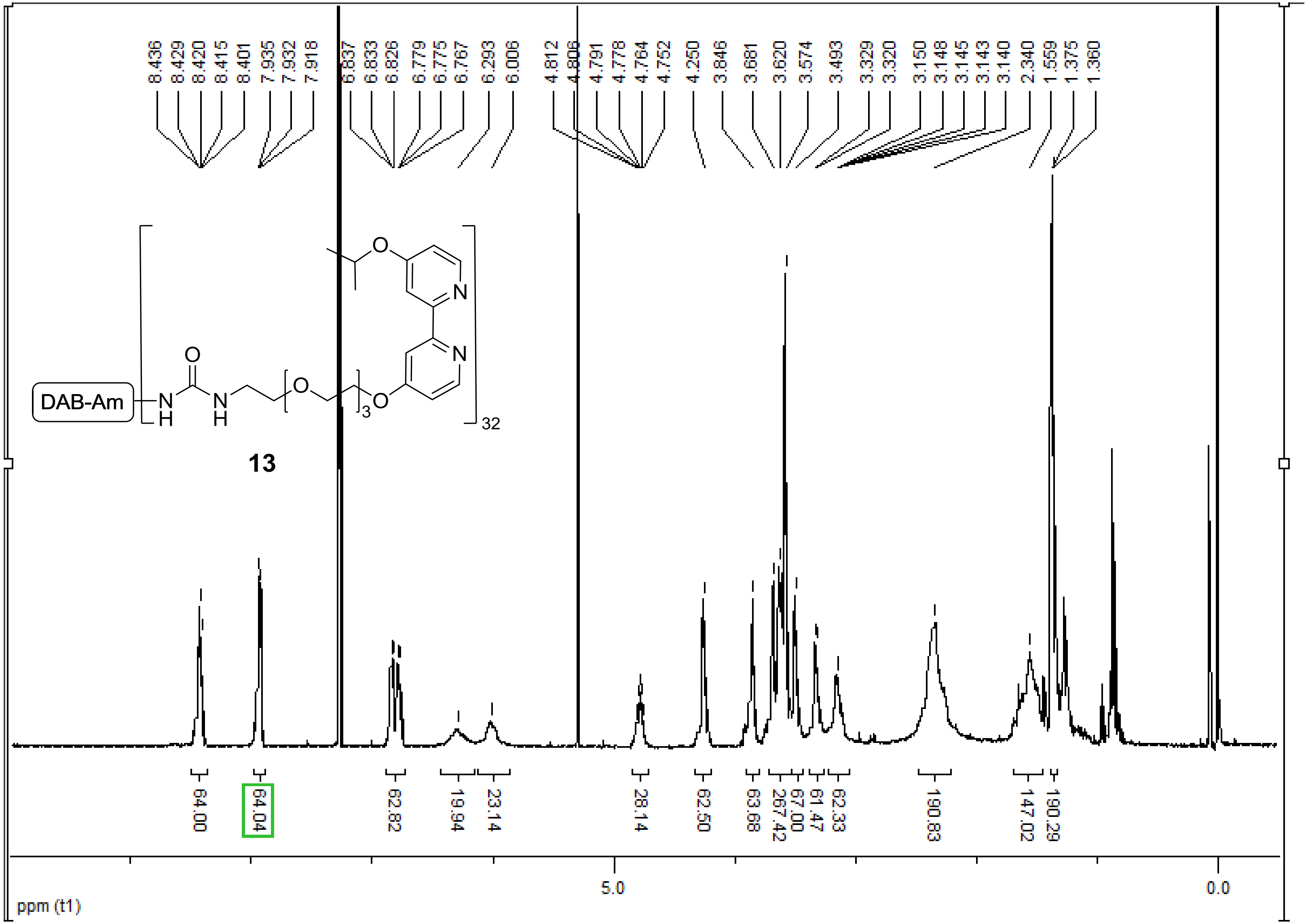

13 ^{13}C NMR

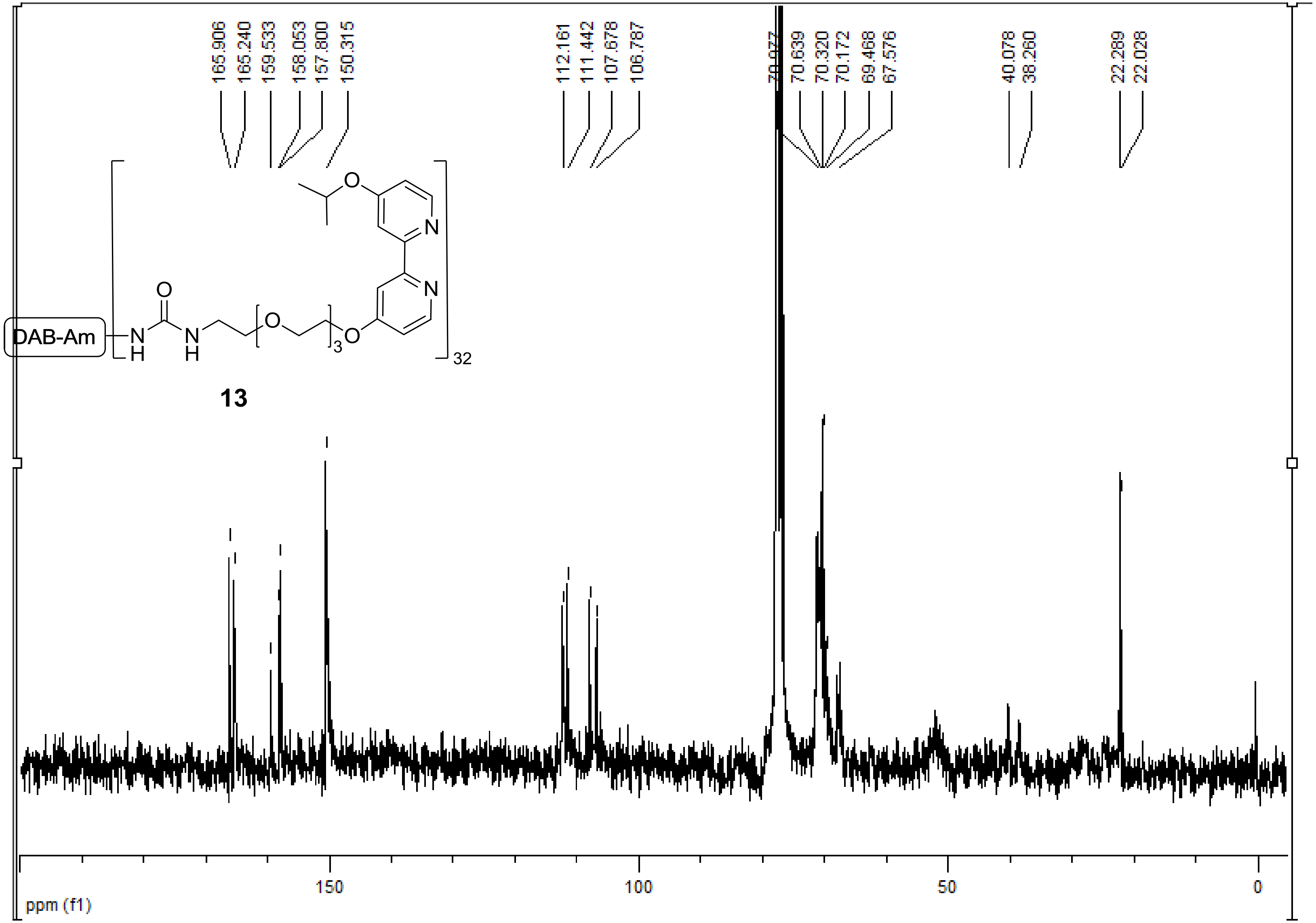
165.906
165.240
159.533
158.053
157.800
150.315
112.161
111.442
107.678
106.787
70.639
70.320
70.172
69.468
67.576
40.078
38.260
22.289
22.028
DAB-Am
O
N
H
3
32
13
150
100
50
0
ppm (f1)

14 ^{1}H NMR

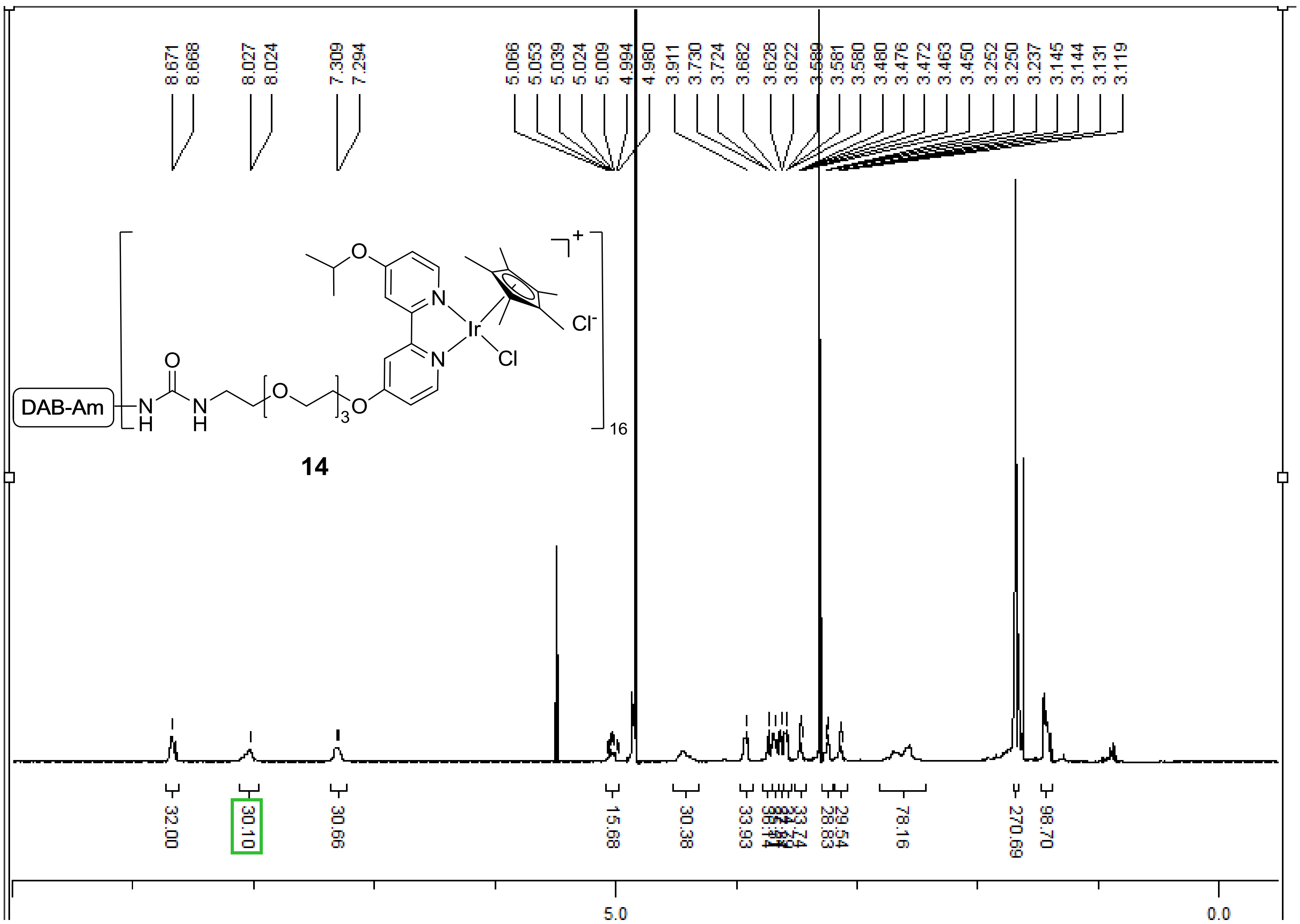

8.671
8.668
8.027
8.024
7.309
7.294
5.066
5.053
5.039
5.024
5.009
4.994
4.980
3.911
3.730
3.724
3.682
3.628
3.622
3.581
3.580
3.480
3.476
3.472
3.463
3.450
3.252
3.250
3.237
3.145
3.144
3.131
3.119
DAB-Am
14
32.00
30.10
30.66
15.68
30.38
33.93
33.74
28.83
29.54
78.16
270.69
98.70
5.0
0.0

14 ^{13}C NMR

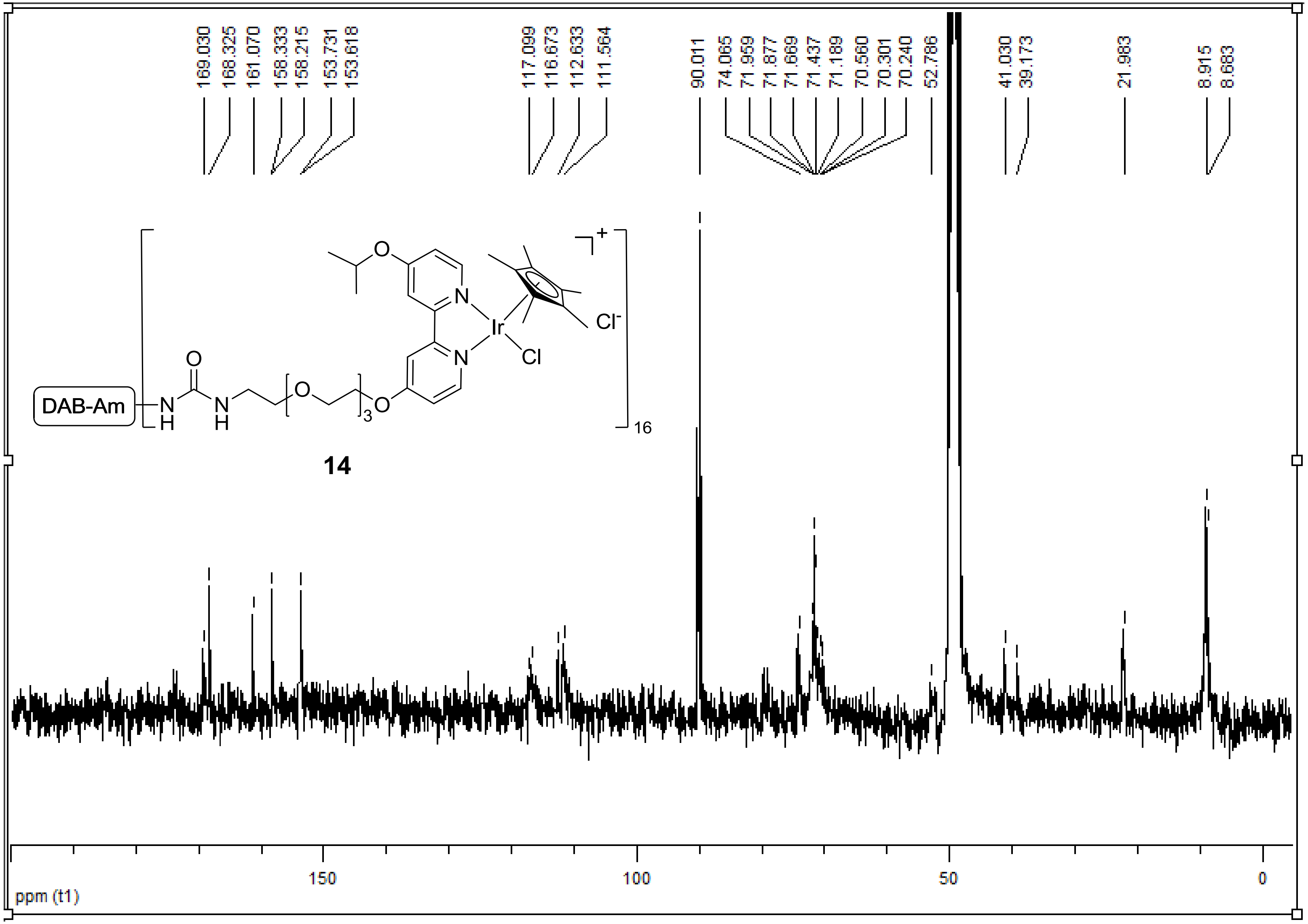

169.030
168.325
161.070
158.333
158.215
153.731
153.618
117.099
116.673
112.633
111.564
90.011
74.065
71.959
71.877
71.669
71.437
71.189
70.560
70.301
70.240
52.786
41.030
39.173
21.983
8.915
8.683
DAB-Am
N
H
O
3
Ir
Cl
Cl-
+
16
14
150
100
50
0
ppm (t1)

15 ^{1}H NMR

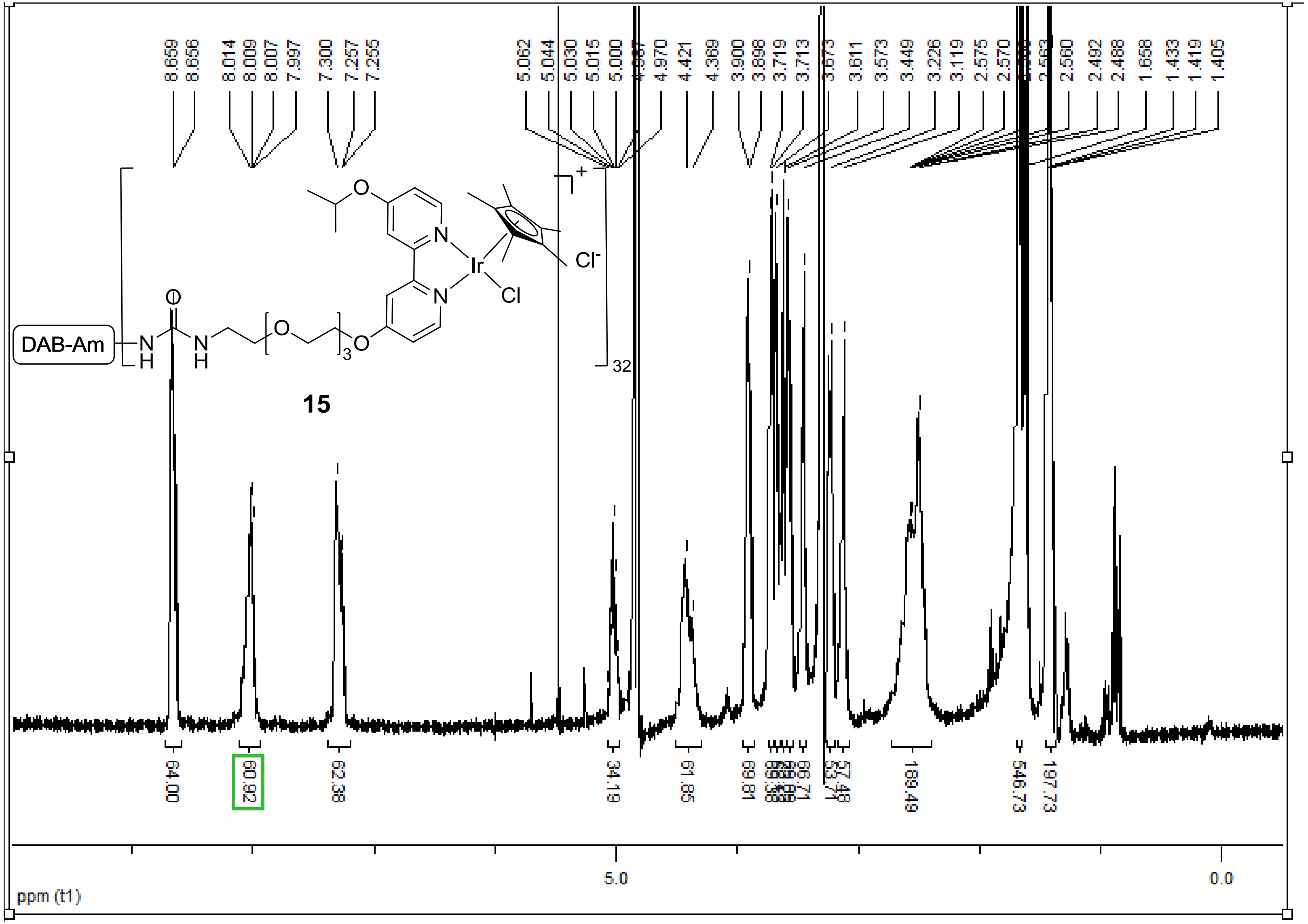

15 ^{13}C NMR

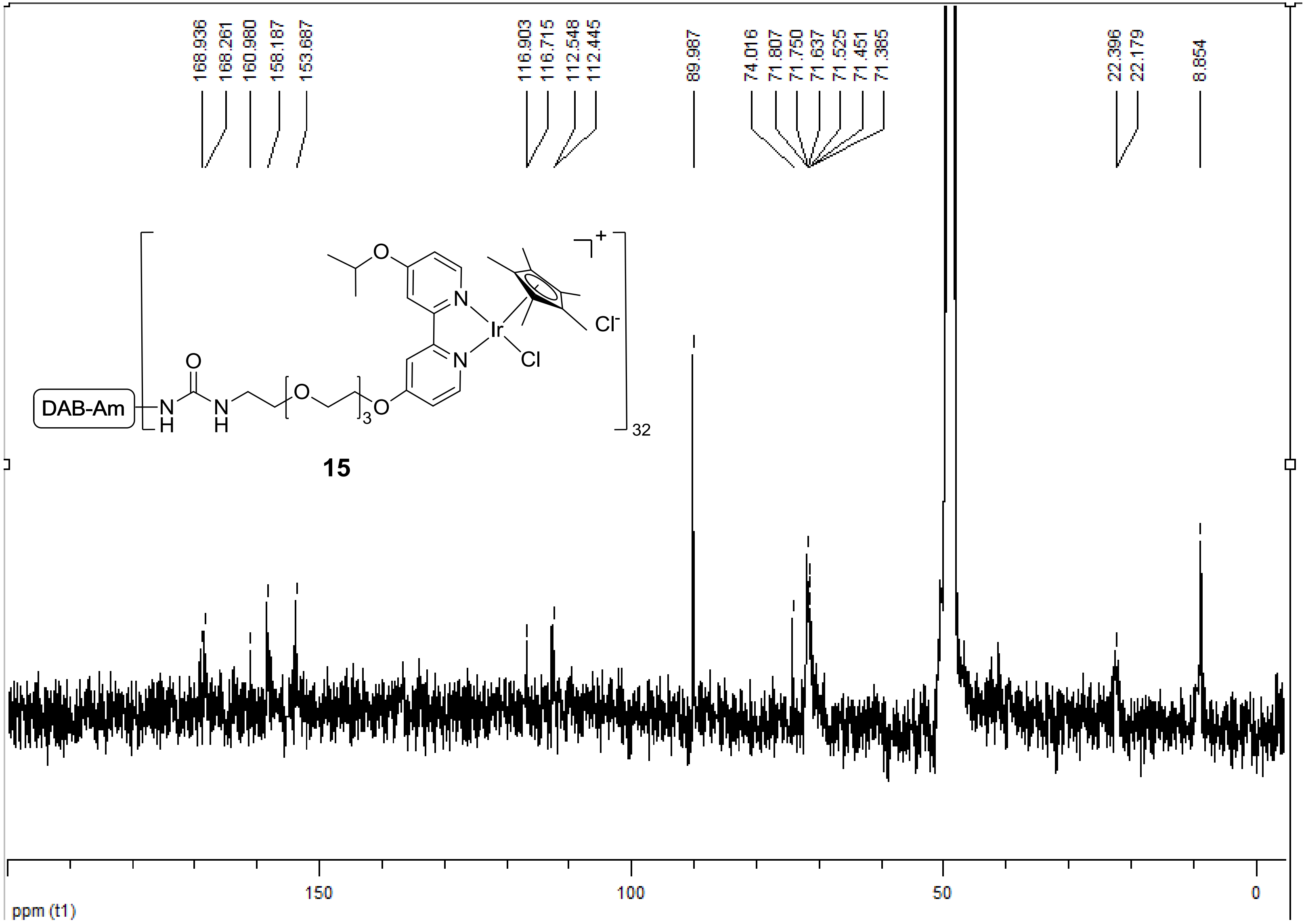
168.936
168.261
160.980
158.187
153.687
116.903
116.715
112.548
112.445
89.987
74.016
71.807
71.750
71.637
71.525
71.451
71.385
22.396
22.179
8.854
DAB-Am
N
H
O
N
H
O
3
O
N
N
Ir
Cl
Cl⁻
+
32
15
150
100
50
0
ppm (t1)

16 ^{1}H NMR

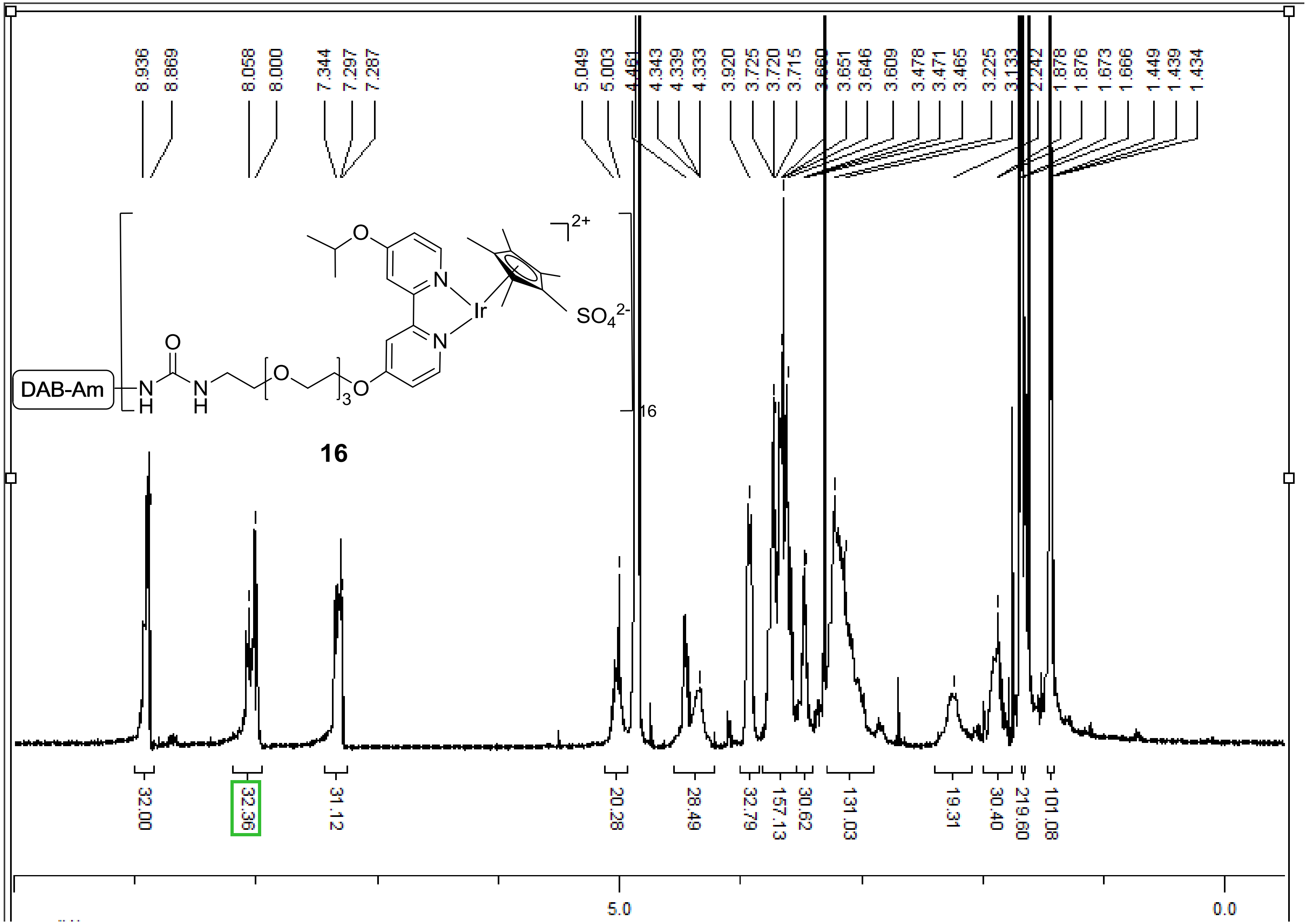

8.936
8.869
8.058
8.000
7.344
7.297
7.287
5.049
5.003
4.461
4.343
4.339
4.333
3.920
3.725
3.720
3.715
3.660
3.651
3.646
3.609
3.478
3.471
3.465
3.225
3.133
2.242
1.878
1.876
1.673
1.666
1.449
1.439
1.434
DAB-Am
16
32.00
32.36
31.12
20.28
28.49
32.79
157.13
30.62
131.03
19.31
30.40
219.60
101.08
5.0
0.0

16 ^{13}C NMR

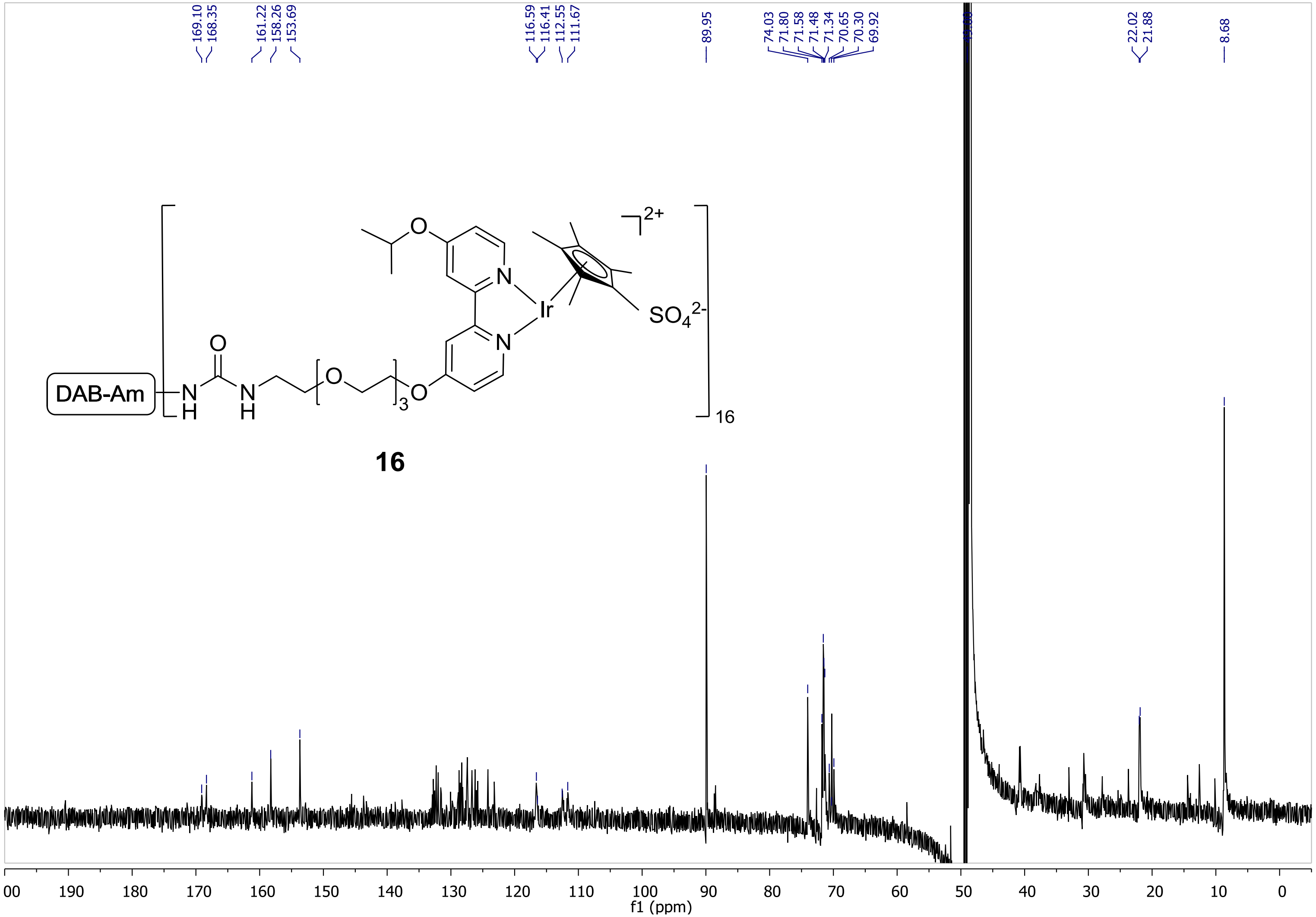

169.10
168.35
161.22
158.26
153.69
116.59
116.41
112.55
111.67
89.95
74.03
71.80
71.58
71.48
71.34
70.65
70.30
69.92
22.02
21.88
8.68
DAB-Am
16
f1 (ppm)

17 ^{1}H NMR

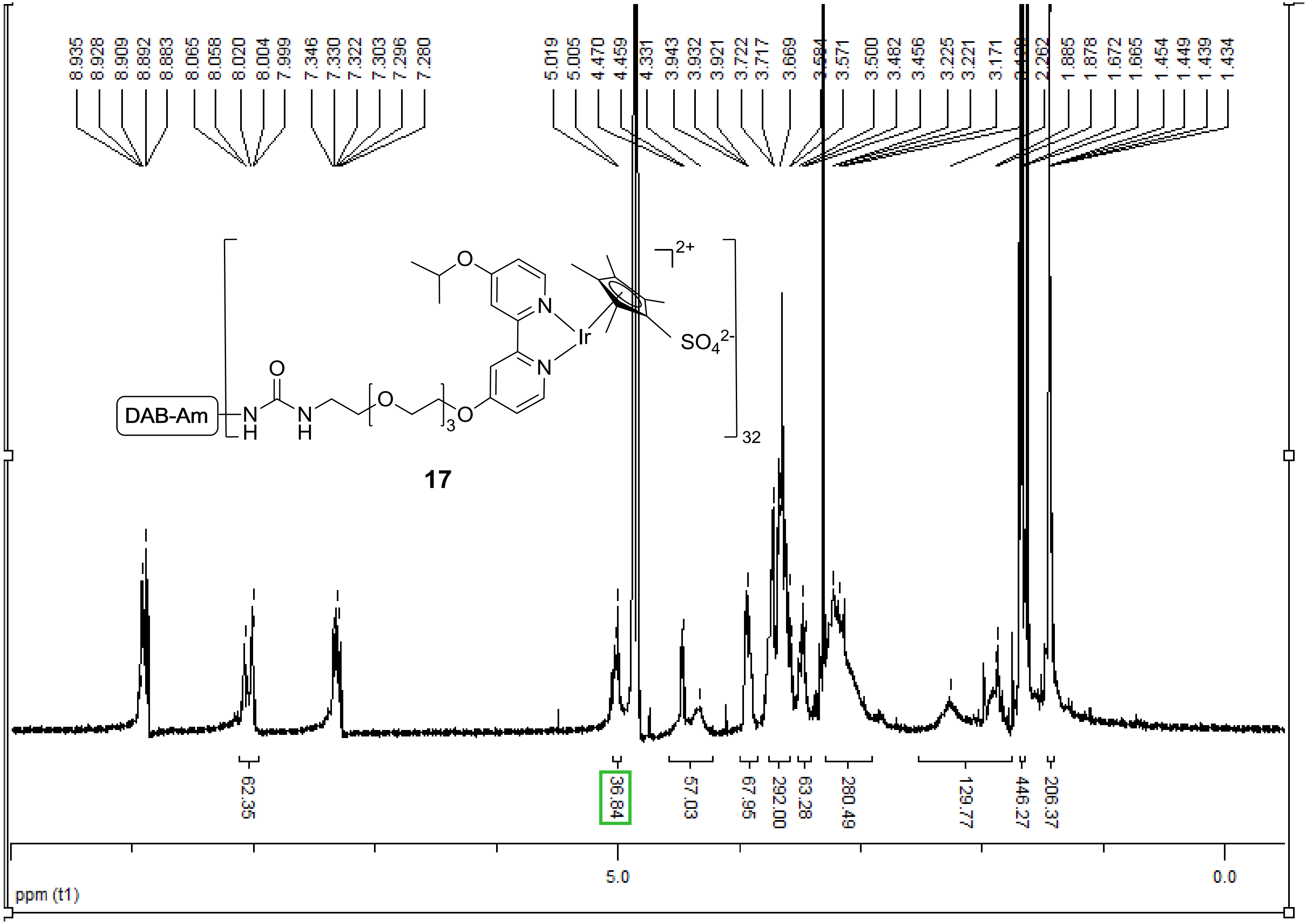

DAB-Am
2+
SO4 2-
32
Ir
17
ppm (t1)
5.0
0.0
62.35
36.84
57.03
67.95
292.00
63.28
280.49
129.77
446.27
206.37

17 ^{13}C NMR

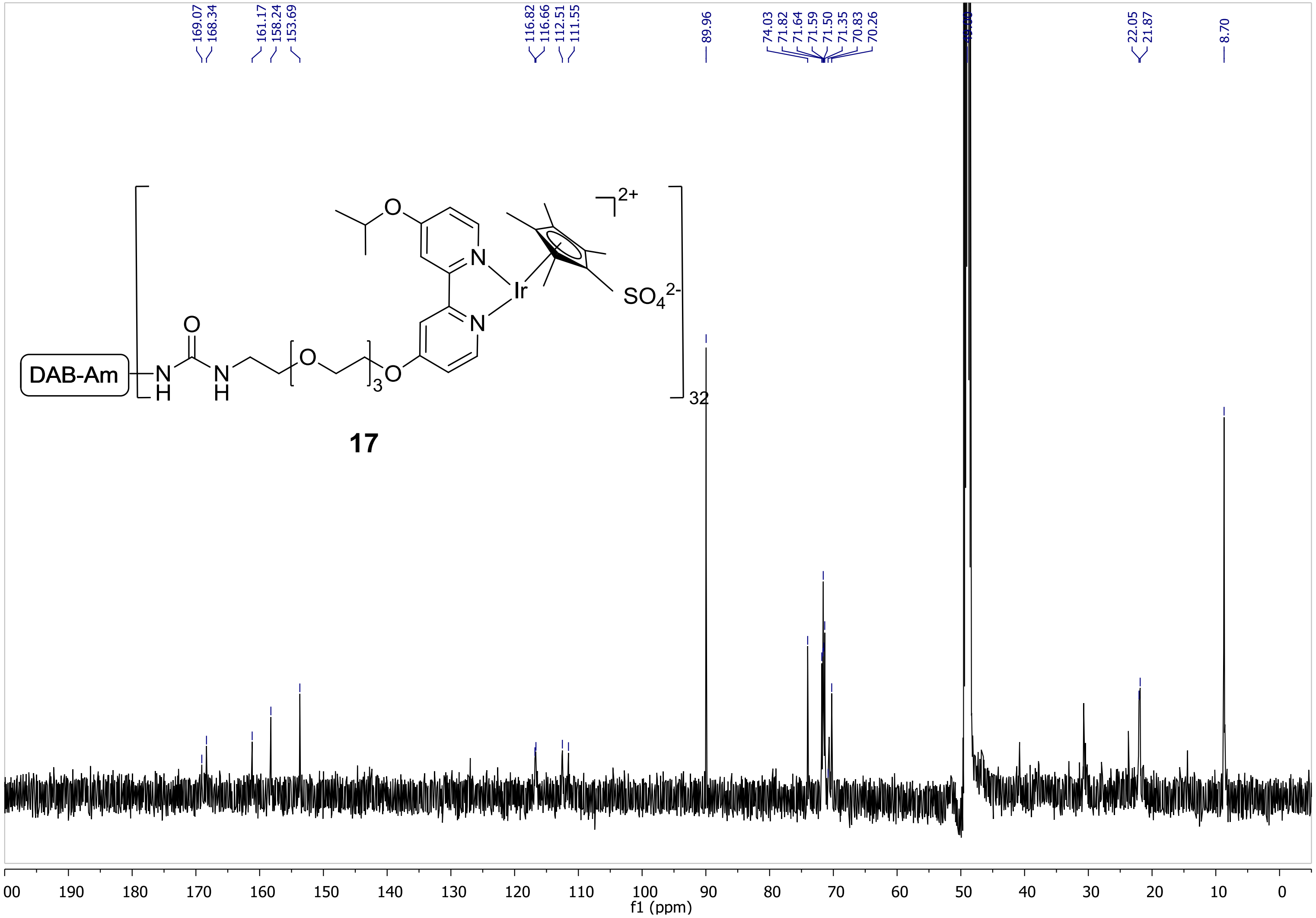

169.07
168.34
161.17
158.24
153.69
116.82
116.66
112.51
111.55
89.96
74.03
71.82
71.64
71.59
71.50
71.35
70.83
70.26
22.05
21.87
8.70
DAB-Am
17
SO4 2-
Ir
f1 (ppm)

19 ^{1}H NMR

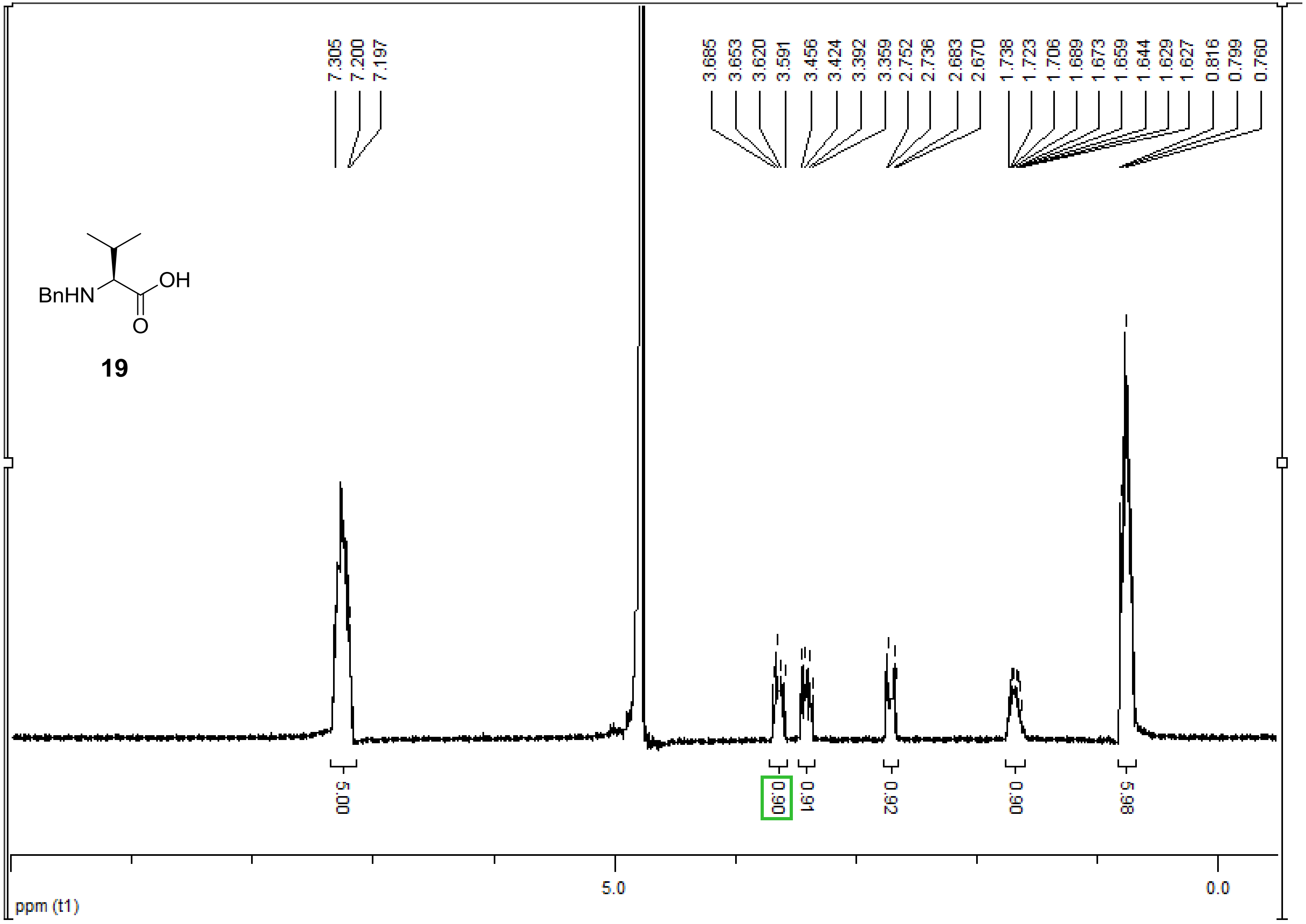

BnHN
OH
O
19
7.305
7.200
7.197
3.685
3.653
3.620
3.591
3.456
3.424
3.392
3.359
2.752
2.736
2.683
2.670
1.738
1.723
1.706
1.689
1.673
1.659
1.644
1.629
1.627
0.816
0.799
0.760
5.00
0.90
0.91
0.92
0.90
5.98
5.0
0.0
ppm (t1)

19 ^{13}C NMR

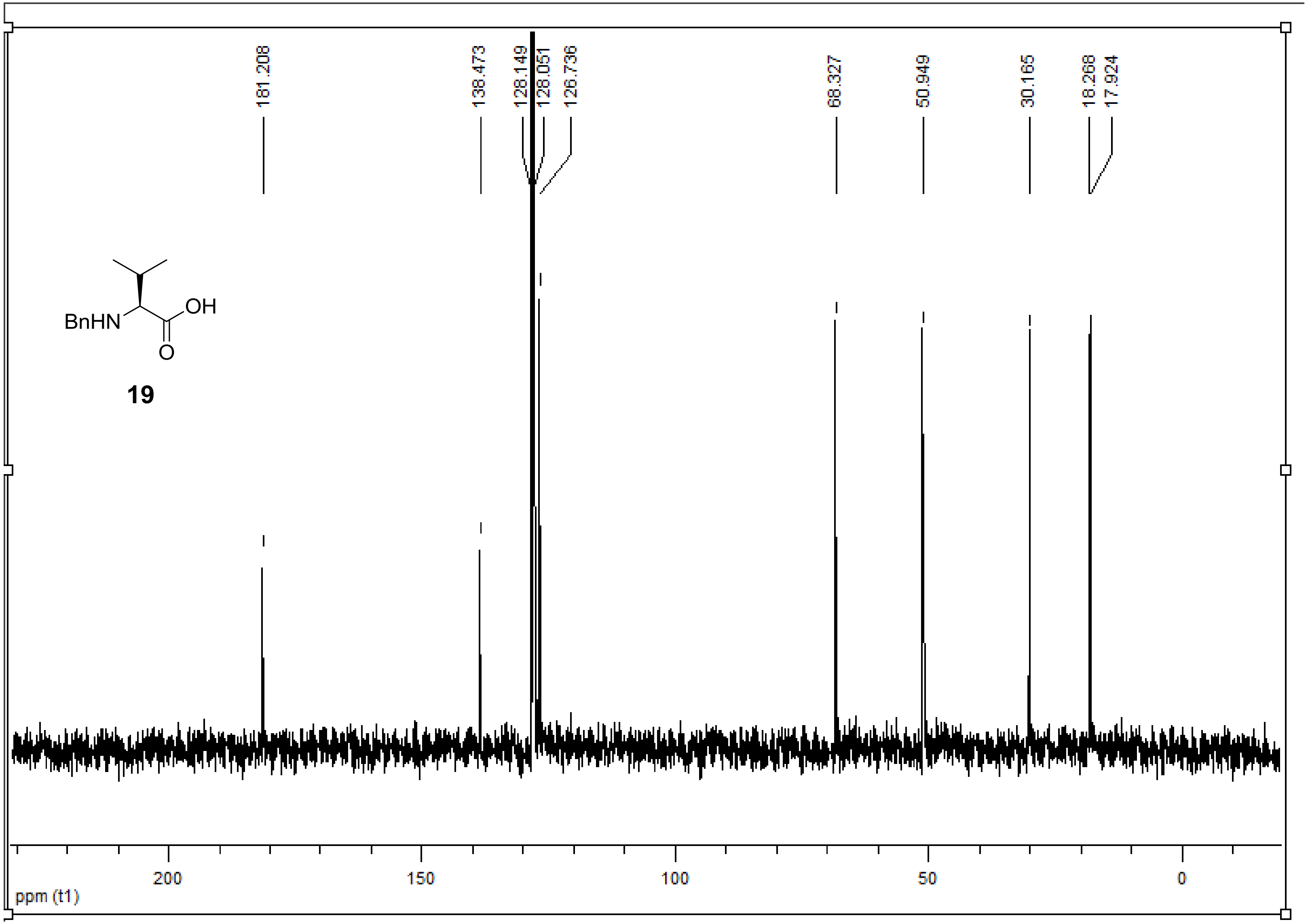

181.208
138.473
128.149
128.051
126.736
68.327
50.949
30.165
18.268
17.924
BnHN
OH
O
19
200
150
100
50
0
ppm (t1)
